# Post-assembly Plasmid Amplification for Increased Transformation Yields in *E. coli* and *S. cerevisiae*

**DOI:** 10.1021/cbe.4c00115

**Published:** 2024-11-18

**Authors:** Thomas Fryer, Darian S. Wolff, Max D. Overath, Elena Schäfer, Andreas H. Laustsen, Timothy P. Jenkins, Carsten Andersen

**Affiliations:** † Department of Biotechnology and Biomedicine, Technical University of Denmark, Søltofts Plads 239, Lyngby, Hovedstaden DK 2800, Denmark; ‡ Department of Molecular Discovery, R&D, Novozymes A/S, Bagsvaerd, Hovedstaden DK 2880, Denmark; § Department of Biochemistry, University of Cambridge, Cambridge CB2 1GA, United Kingdom

**Keywords:** Directed evolution, DNA library preparation, rolling circle amplification, transformation efficiency, *in vivo* homology directed assembly

## Abstract

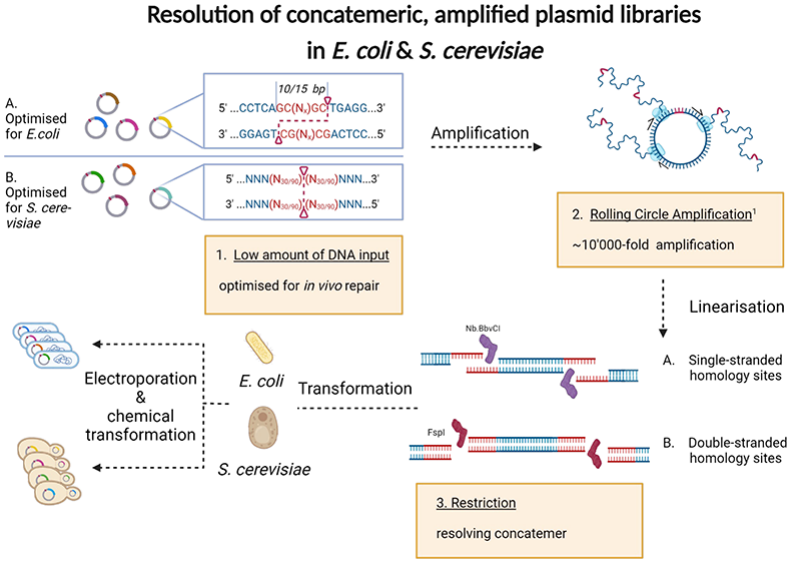

Many biological disciplines rely upon the transformation of host cells with heterologous DNA to edit, engineer, or examine biological phenotypes. Transformation of model cell strains (*Escherichia coli*) under model conditions (electroporation of circular supercoiled plasmid DNA; typically pUC19) can achieve >10^10^ transformants/μg DNA. Yet outside of these conditions, e.g., work with relaxed plasmid DNA from *in vitro* assembly reactions (cloned DNA) or nonmodel organisms, the efficiency of transformation can drop by multiple orders of magnitude. Overcoming these inefficiencies requires cost- and time-intensive processes, such as generating large quantities of appropriately formatted input DNA or transforming many aliquots of cells in parallel. We sought to simplify the generation of large quantities of appropriately formatted input cloned DNA by using rolling circle amplification (RCA) and treatment with specific endonucleases to generate an efficiently transformable linear DNA product for *in vivo* circularization in host cells. We achieved an over 6500-fold increase in the yield of input DNA, and demonstrate that the use of a nicking endonuclease to generate homologous single-stranded ends increases the efficiency of *E. coli* chemical transformation compared to both linear DNA with double-stranded homologous ends and circular Golden-Gate assembly products. Meanwhile, the use of a restriction endonuclease to generate linear DNA with double-stranded homologous ends increases the efficiency of chemical and electrotransformation of *Saccharomyces cerevisiae*. Importantly, we also optimized the process such that both RCA and endonuclease treatment occur efficiently in the same buffer, streamlining the workflow and reducing product loss through purification steps. We expect that our approach could have utility beyond *E. coli* and *S. cerevisiae* and be applicable to areas such as directed evolution, genome engineering, and the manipulation of alternative organisms with even poorer transformation efficiencies.

## Introduction

The manipulation of DNA and its subsequent insertion into a desired host cell are a bedrock of modern biotechnology. This ability has advanced both basic science (e.g., the human genome project, metagenomics, gene, and protein function assays) and applied science (e.g., the directed evolution of proteins for a desired functionality and the production of proteins and small molecules via fermentation and biocatalytic processes). To achieve DNA transfer into microorganisms, there are three main routes: conjugation, transduction, and transformation, in which DNA is transferred cell-to-cell, virus-to-cell, or extracellular DNA-to-cell, respectively. Transformation is the most routinely used DNA transfer mechanism in laboratory settings due to its relative ease (eschewing any need for viral packaging reactions^1^ or coculturing of conjugative cells^2^) and historical precedent, in which protocols have been established for many different cell types across all kingdoms of life. In particular, many protocols have been established for two of the most common organisms used in biotechnology, *Escherichia coli*(3) and *Saccharomyces cerevisiae*,^4^ that are broadly separable into either chemically- or electro-competent methods. Briefly, chemically competent approaches typically rely on washing in mixtures of salts followed by subsequent incubation with exogenous DNA that is induced to pass through the cell membrane by a heat-shock step, while electrocompetent approaches wash cells to remove any ions and then use exposure to electrical current to drive exogenous DNA through the cellular membrane.^5^ Despite the vast number of reported protocols typically fewer than 5% of cells are transformed by exogenous DNA^6^ even under model conditions (e.g., efficient cell type and strain coupled to small quantities of supercoiled small plasmid DNA, as demonstrated in our hands by <3% of electrocompetent cells, and <1% of chemically competent cells using circular, supercoiled DNA in our own hands Figure 2D). When working with nonmodel conditions or cell types transformation efficiencies drop severely, for instance, *in vitro* DNA cloning preparations (using nanogram-microgram quantities of cloned relaxed circular DNA) often results in 10–100-fold loss in efficiency (as seen in our own data, Figure 2D).

In order to overcome limited transformation efficiencies, researchers typically scaled up the process by transforming multiple aliquots of cells using as much DNA as possible. While multiple aliquots of cells can either be purchased (at significant cost) or prepared in-house with relative ease, the preparation of large quantities (10–100 μg) of DNA is more difficult. To achieve large quantities of assembled DNA, one can either scale-up the assembly reactions or amplify the assembled product in a way that it is suitable for transformation into host cells. There are now many different ways to assemble DNA beyond the traditional restriction-ligation approach, many of which offer the benefit of “scarless” assembly, i.e., the absence of any undesired sequence in the final product. The most common scarless approaches are Gibson assembly^7^ and Golden-Gate assembly^8^ encountered in the form of commercial kits, which cause the scale-up of library assemblies to quickly become economically infeasible for many scientists with limited access to equipment and resources as pointed out by Xia et al.^9^ Much work has gone into noncommercial derivatives^10^ or alternatives,^9,11^ and perhaps the most established noncommercial scarless cloning approach utilizes the native ability of multiple cell types to *in vivo* repair linear DNA into circular plasmid DNA. While it is widely known that *S. cerevisiae* is transformable by linear DNA with homologous ends,^12^ it is often overlooked that *E. coli* possesses the same ability^13^ (albeit likely occurring through a different nonrecombination-based pathway). As these approaches unite the plasmid assembly and transformation steps in a cost-free manner, they are particularly scalable and therefore suitable for large library generation. Thus, a method to generate large quantities of DNA in a suitable format (i.e., linear DNA) is required. Much work on amplifying DNA from small-scale assemblies has already been carried out, typically focusing on rolling circle amplification (RCA) due to its ability to massively amplify circular DNA in an isothermal manner. The product of RCA (highly branched concatomers) is however not directly suitable for transformation and must be resolved into monomeric units, achieved in the literature through digestion with restriction enzymes and subsequent large-volume low concentration religation^6^ or Cre recombinase activity.^14^ While both approaches are successful, they are either cumbersome or require nonstandard reagents, and result in circular DNA molecules that are poorly suited to transformation into hosts such as *S. cerevisiae*. During manuscript preparation another elegant approach was developed that utilizes a one-pot RCA reaction coupled to simultaneous restriction and ligation, that generates large quantities of circular DNA as opposed to our linear DNA.^15^ To best deliver a method that can easily generate large quantities of DNA for transformation into different cell types, we thus sought to combine features from multiple different approaches that would result in large quantities of linear DNA with appropriate presentation of homologous ends for efficient *in vivo* circularisation. Notably, we utilized RCA to amplify a small-scale Golden-Gate assembly reaction ∼10,000-fold, generating ∼100 μg of product, followed by nicking endonuclease (Nb. BbvCI) treatment (*E. coli* workflow) to generate linear DNA with single-stranded homologous ends or blunt-ended restriction endonuclease (FspI) treatment (*S. cerevisiae* workflow) to generate linear DNA with double-stranded homologous ends. Subsequently, these linear DNA molecules can be efficiently transformed into the appropriate cell types thus enabling, if repeated using the large quantities of DNA available, substantially increased numbers of transformants to be achieved.

## Experimental Section

### Plasmids and Primers

Plasmids employed for transformations in *E. coli* are derivatives of the plasmid “PF-Nbb102-CAM”, a VHH encoding plasmid (phagemid-derived from the commercially available pADL22c) with chloramphenicol resistance gene and have been modified by amplification of designed primers ordered from IDT (Integrated DNA Technologies, Inc.). Similarly, *S. cerevisiae* transformation tested were conducted by employing derivates of the plasmid “pCT anti-GFP” a yeast display vector containing an anti-GFP VHH

Sequences are listed in Table S1.

### DNA Manipulation

#### Nickase Site Insertion

In the phagemid context nickase sites were added through PCR amplification using Q5 DNA polymerase and appropriate DNA primer pairs (F NbBbvCI 10bp ‘gcaggtttgc*tgagg*CCCGACTGGAAAGCGGGC’ with R NbBvCI 10 bp ‘gcaaacctgc*tgagg*GTCGTGCCAGGGCATCCC’ and F NbBbvCI 15 bp ‘gcacgacaggtttgc*tgagg*CCCGACTGGAAAGCGGGC’ with R NbBbvCI 15 bp ‘gcaaacctgtcgtgc*tgagg*CCAGGGCATCCCTCCTTTCA’). In upper case is the annealing site, italics the Nb.BbvCI site, and underlines the homology region for *in vivo* assembly. PCR product was DpnI treated and purified using homemade SPRI beads. Transformation of these linear DNA products results in plasmids containing the appropriate nickase cassettes through *in vivo* assembly.

Similarly the yeast plasmid nickase cassettes were inserted through amplification of the pCT anti-GFP backbone with appropriate DNA primer pairs followed by transformation into *E. coli* for *in vivo* assembly (F Nick 30 bp ‘tggccgattcattaatgcagttt*gctgagg*CTCCAATTCGCCCTATAGTG’ with R Nick 30 bp ‘ctgcattaatgaatcggccacct*gctgagg*CTCAATTCTCTTAGGATTCGATTC’ and F Nick 90 bp ‘tacactattctattggaatcttaatcattctggccgattcattaatgcagttt*gctgagg*CTCCAATTCGCCCTATAGTG’ with R Nick 90 bp ‘gattccaatagaatagtgtataaattatttatcttgaaaggagggatgcccct*gctgagg*CTCAATTCTCTTAGGATTCGATTC’). In upper case is the annealing site, italics the Nb.BbvCI site, and underlined the homology region for *in vivo* assembly. The double-stranded homology cassettes were built on top of the pCT anti-GFP plasmids already containing the 30 bp or 90 bp nickase cassettes, first the appropriate plasmids were amplified with (F pCT BsaI ‘gagtag*ggtctc*cTGAGGCTCCAATTCGCC’ and R pCT BsaI ‘gaggat*ggtctc*aCTCAGCAAACTGCATTAATGAATCGGCCA’), second the 30 bp/90bp cassettes were created by mixing the two appropriate primers alone in a PCR reaction (F FspI cassette 30 bp ‘gaggta*ggtctc*attgcgcaggTGGCCGATTCATTAATGCAG’ with R FspI cassette 30 bp ‘gaggat*ggtctc*actcagcaaaCTGCATTAATGAATCGGCCA’ and F FspI cassette 90 bp ‘gagat*ggtctc*attgcgcaggggcatccctcctttcaagataaataatttaTACACTATTCTATTGGAATC’ with R FspI cassette 90 bp ‘gagat*ggtctc*actcagcaaactgcattaatgaatcggccagaatgattaaGATTCCAATAGAATAGTGTA’. In upper case is the annealing site, and italics the BsaI site. The products were then appropriately mixed in a Golden-Gate assembly reaction using BsaI-HFv2 (NEB: R3733L) and T4 DNA ligase (NEB: M0202L) and transformed into *E. coli*.

#### Amplification

Initially, while seeking to optimize the conditions for amplification, we tested two different phi29 polymerases: NEB phi29 polymerase (New England Biolabs, catalogue no.: M0269L) and EquiPhi29 DNA Polymerase (ThermoFisher Scientific, catalogue no.: A39390). Employing both polymerases, we assessed three randomized hexamer primers (random DNA hexamers (NNNNNN), random DNA hexamers with 3x phosphorothioate bonds (N*N*N*NNN), and random RNA hexamers) and one defined primer pair, which was known to anneal to specific sites of the plasmid in question (forward-PD116 “CATGACCAAAATCCCTTAAC” and reverse-PD117 “CATGAGCGGATACATATTTG”).

In general, <1 ng of DNA of interest was pipetted to a mix of 1x rCutsmart buffer (New England Biolabs), 100 μM of primer, and nuclease-free water. The sample was denatured at 95 °C for 3 min and immediately placed on ice for another 3–5 min. For the amplification step, 1 mM dNTP, 1× rCutsmart buffer, Milli-Q water, and 10 U of the respective ϕ29 DNA polymerase were added to a final reaction volume of 20 μL.

For amplifications facilitated by EquiPhi29 DNA polymerase, we added 1 mM DTT and incubated the reaction mix at 45 °C for 3 h. Reaction mixes containing NEB Phi29 0.1 mg/mL recombinant albumin were incubated for 16 h at 30 °C. Both polymerases were heat inactivated by heating the sample at 65 C for 10 min.

#### Linearization

To resolve the concatemeric structure, 1 μg of RCA product was restricted by employing 10 U nicking endonuclease Nb.BbvCI (New England Biolabs, R0631L) in a 50 μL reaction volume with 1× rCutsmart buffer for 1 h at 37 °C, followed by a heat inactivation step at 80 °C for 20 min. In case of double-stranded linear DNA applied in transformation experiments with *S. cerevisiae*, the RCA product was resolved to linear monomers by restriction using the endonuclease FspI (New England Biolabs, R0135L) under similar conditions.

All DNA concentration measurements were conducted with a Qubit broad range assay kit (ThermoFisher Scientific Inc.)

#### Golden Gate Assembly

Golden Gate assemblies were performed in 20 μL of reactions containing 160 U T4 DNA ligase (NEB: M0202L), and 24 U BsaI-HFv2 (NEB: R3733L), 2 μL of T4 DNA Ligase Buffer (10x), 100 ng of PCR-amplified and DpnI-treated vector DNA, a 3-fold molar excess of similarly PCR-amplified and DpnI-treated insert DNA and Milli-Q water. The reaction mix was incubated for 30 cycles of 5 min incubations at alternating temperatures of 37 and 16 °C, followed by a final 65 °C incubation for 10 min. Golden Gate assemblies prior to RCA were two-fragment reactions (one insert and one vector). The resulting product was purified by using homemade solid-phase reversible immobilization (SPRI) beads.

#### Purification

To ensure a low salt concentration and, thus, a low ionic strength, all DNA was purified prior to any transformation (chemical and electroporation) by employing solid-phase reversible immobilization (SPRI) beads. The commercially available bead solution Sera-Mag Carboxylate-Modified [E3] Magnetic Particles (Cytiva, 44152105050250) was diluted 1:50 (v/v) in deionized water containing 20% PEG_8000_ and 2.5 M NaCl and applied in a 1:1 volumetric ratio to the DNA sample, rigorously mixed, and incubated for 1 min. Next, the beads were captured on a magnetic rack, and the supernatant was discarded. While immobilized on the magnetic rack, the same volume of SPRI wash buffer (70% (v/v) ethanol containing 0.05% Tween-20 (v/v)) as the previously discarded supernatant was added to the captured beads. The magnetic rack was repeatedly, but gently, inverted and the supernatant removed. The washing procedure was repeated three times, and any residual SPRI wash buffer was removed by briefly centrifuging the tubes, and pipetting off any remnant liquid. DNA was then eluted by resuspending the beads in a desired volume of Milli-Q water, and then collecting the supernatant after bead capture on a magnetic rack.

### Production of Competent Cells

#### Preparation of Electrocompetent *E. coli* Strains XL1-Blue and BL21 (DE3)

Five milliliters of LB medium (including 15 mg/mL tetracycline for XL1-Blue) was inoculated from respective glycerol stocks and grown overnight at 37 C and 200–250 rpm. Next, 500 mL of SOB (pH 7.5, 5 g/L yeast extract, 20 g/L tryptone/peptone, 0.584 g/L NaCl, 0.186 g/L KCl, 2.4 g/L MgSO4) were added to a 2.5 L shake flask and inoculated by cells from the previous culture to an initial OD of 0.007. The culture was grown slowly at 20 °C and 250 rpm for 42 h to a final OD of 0.6.

Subsequently, the culture was stored on ice, pelleted (2500*g*, 4 °C, 9/12/15 min), and washed with ice-cold Milli-Q water three times, while steadily reducing the resuspension volume (250/50/25 mL) and adding 10% final concentration of glycerol from the second wash step onward. Lastly, pellets were resuspended in 5 mL of ice-cold Milli-Q water with 20% glycerol in case the cells were stored in −80 °C or without glycerol if directly used for transformation.

#### Preparation of Chemically Competent *E. coli* Strains XL1-Blue, and BW25113

##### Adjusted Inoue Protocol

Cultures from *E. coli* XL1-Blue were grown as previously described for the preparation of electrocompetent *E. coli* and harvested at an OD between 0.4 and 0.6. The cultures were pelleted (4000*g*, 10 min, and 4 °C) and resuspended in sterilized 250 mL TB buffer (pH 6.7 (adjusted with KOH), PIPES 3.021 g/L, CaCl_2_·2H_2_O 11.025 g/L, KCl 18.637 g/L, MnCl_2_·4H_2_O 10.885 g/L). Pelleting was repeated after storing the resuspended mixture on ice for 10 min. Next, cells were resuspended in 40 mL TB buffer and 3 mL DMSO, gently mixed, and stored on ice for another 10 min. Subsequently, the resuspended cells were distributed in aliquots of 100 μL and either directly used or stored at −80 °C.

##### TSS-HI (Transformation Storage Solution Optimized by Hannahan and Inoue Method)

To test further protocols for chemically competent *E. coli*, we took inspiration from Yang et al. and tested the strain BW25113^16^ with their improved TSS-HI protocol.^17^ Four mL portion of LB medium was inoculated, and cells were grown overnight at 250 rpm and 37 °C. Here 1% (40 μL) of the culture was transferred to fresh 50 mL of LB and grown to an OD of 0.5. Subsequently, the cells were stored on ice for 10 min, centrifuged at 4 °C and 4000*g*, and resuspended in 1 mL of chilled (0 °C) TSS-HI.

##### Mix & Go *E. coli* Transformation Kit (T3001)

Benchmarking the chemical competence protocols for the *E. coli* strain XL1-Blue, we tested the commercial transformation kit Mix & Go *E. coli* Transformation Kit (T3001) available at (zymoresearch.com) and followed the general guidance (V.1.18) including Notes for High Efficiency Transformation.

#### Chemical Competent *S. cerevisiae* Strain EBY100

*S. cerevisiae* strain EBY100 cells were made competent based on the “Li/Ac protocol” of Gietz and Schiestl.^4^ Deviating from the protocol, we inoculated 100 mL of the second culture with a final OD of 0.25. Cells were harvested after 6 h of incubation at an OD of 0.95, representing a total cell titer of 9.5 × 10^8^ cells in 100 mL culture volume.

#### Electrocompetent *S. cerevisiae* Strain EBY100

For the preparation of electrocompetent EBY100 cells, we followed the protocol of Benatuil et al.^18,19^ In short, we grew *S. cerevisiae* cells (EBY100) overnight to stationary phase (OD ∼ 3) in YPD media (10 g/L yeast nitrogen base, 20 g/L Peptone and 20 g/L D-(+)-Glucose) on a platform shaker at 250 rpm and 30 °C. The next morning, 500 mL of fresh YPD media was inoculated using an aliquot of the overnight culture to an initial OD of 0.3 and subsequently incubated at 30 °C and 225 rpm until OD was 1.6. Cells were collected by centrifugation at 3000 rpm for 3 min at 4°C, the samples were washed twice with 250 mL of ice-cold water and once with 250 mL of ice-cold electroporation buffer (1 M Sorbitol/1 mM CaCl_2_). Next, cells were resuspended in 100 mL of 0.1 M LiAc/10 mM DTT and shaken at 230 rpm in a culture flask for 30 min at 30 °C. Once again, cells were collected, washed once with 250 mL of ice-cold electroporation buffer, and resuspended in 500 to 1000 μL of electroporation buffer to reach a final volume of 4.0 mL. This corresponds to approximately 8 × 10^9^ cells in total and is sufficient for 10 electroporations at 400 μL total volume per 0.2-m gap cuvette. The cells were kept on ice until electroporation.

### Transformation

#### Electroporation of *E. coli* Strains

Aliquots of electrocompetent *E. coli*, directly after preparation or stored at −80 °C, were slowly thawed on ice. Once thawed, the sample DNA (in deionized water) was added to a maximal voluminal ratio of 5%, typically 5 μL or less to 50 μL of cell suspension, gently mixed by pipetting, and incubated for 3–5 min on ice. Next, the chilled DNA/cell suspension was transferred to a prechilled 0.1 cm gap cuvette (BioRad) and electroporated using a Gene Pulse Controller electroporation system (Bio-Rad) at 1.5 kV voltage, 200 Ω resistance, and 25 kF/cm^2^ capacity. After applying the electroshock (typical response time of 4–5 ms), we rescued the cells by adding 1 mL of prewarmed (37 °C) SOC media including 2% Glucose and 5 mM MgCl_2_ into the cuvette, slowly pipetting up- and down, and finally collecting the entire cell suspension in a 1.5 mL reaction tube. The suspension was incubated for 90 min at 37 °C, 750 rpm on a table-top thermoshaker. In the meantime, selective plates (SOC, 2% glucose, and respective antibiotics) were prewarmed to 37 °C. Lastly, the samples were diluted in a 1/10 dilution series and plated as 5 μL spots in triplicates on selective plates.

#### Chemical Transformation of *E. coli* Strains

##### Adjusted Inoue Protocol

1 μL of the DNA sample (in deionized water) was added to 30 μL of cells in PCR reaction tubes on ice and gently mixed. After 30 min of incubation on ice, the cell/DNA suspension was exposed to a 45 s heat shock at 42 °C and immediately chilled on ice for 2 min. The cell suspension was transferred to a 1.5 mL reaction tube containing 1 mL of prewarmed growth media (SOB including 2% (w/v) of D-(+)-Glucose and 5 mM MgCl_2_) and incuabted at 37 °C for 1 h with gentle shaking in a tabletop thermomixer. The cell suspension was plated in a dilution series of 1/10 in 5 μL droplets on selective agar plates.

##### TSS-HI (Transformation Storage Solution Optimized by Hannahan and Inoue Method)

The TSS-HI method developed by Yang et al.^17^ requires a preparation step for the DNA sample prior to the mixture of DNA and cell suspension. The DNA sample was resuspended in 5× KCM (0.5 M KCl, 150 mM CaCl_2_, 250 mM MgCl_2_) and deionized water to a total volume of 25 μL. The DNA reaction mix was transferred, gently mixed, and incubated with 25 μL of cell suspension for 30 min on ice. Similar to the “adjusted Inoue method”, a heat-shock of 45 s at 42 °C was applied before the cells were incubated on ice for two min Lastly, the cells were recovered in a 1 h incubation in 250 μL of prewarmed LB medium and plated as stated previously.

##### Mix & Go *E. coli*

Following the protocol provided with the Mix & Go *E. coli* Transformation Kit & Buffer Set (T3001) and paying special attention to the statements in the chapter “Notes for high transformation efficiency,” the chemical competent cells grown with ZymoBroth were transformed similar to the Inoue method described before, although the heat shock was omitted. Instead, after 30 min of incubation on ice, the cell suspension was transferred to 1.5 mL reaction tubes with 1 mL of prewarmed growth media SOB including 2% (w/v) of D-(+)-Glucose and 5 mM MgCl_2_.

#### Chemical Transformation of *S. cerevisiae* Strain EBY100

Yeast transformations were always conducted directly after the cell preparation. Aliquots of 100 μL of cell suspension were spun down at 13000 x rpm for 30 s and resuspended in 336 μL of transformation mix (0.1 M LiAc, 240 μL of PEG 3350 50% (w/v)) including 0.28 mg/mL single-stranded carrier DNA (Dual Systems, lot no. 6001120319) and the specific DNA sample of interest. Samples were exposed to a subsequent 40 min heat shock at 42 °C and 650 rpm shaking, followed by pelleting and resuspension in 1 mL of sterile Milli-Q water. Samples were diluted in a 1/10 series and plated on selective tryptophan dropout media agar plates.

#### Electroporation of *S. cerevisiae* Strain EBY100

Electroporation of yeast cells followed their preparation immediately. The cell suspension was aliquoted to 370 μL per sample and mixed gently with 30 μL Milli-Q including 8 μg of target DNA. After an incubation not exceeding 5 min the chilled DNA/cell suspension was added to a prechilled 0.2 cm gap cuvette (BioRad) and electroporated using a Gene Pulse Controller electroporation system (Bio-Rad) at 2.5 kV voltage, 200 Ω resistance, and 25 kF/cm^2^ capacity. After applying the electroshock (typical response time around 3.8–4.5 ms), cells were rescued by adding 1 mL of prewarmed (30 °C) 1:1 mix of 1 M sorbitol: YPD media into the cuvette, pipetting slowly up- and down, and then transferring to a 50 mL centrifuge tube, which already contained 6 mL prewarmed 1:1 mix of 1 M sorbitol:YPD media. The rescue procedure was repeated with a fresh 1 mL, yielding a total of 8 mL culture. The suspension was incubated for 60 min, at 30 °C, 225 rpm. In the meantime, selective plates (SD (-Trp) + 2% Glucose including 10 mg/mL chloramphenicol) were prewarmed to 30 °C. Lastly, we diluted the samples in a 1/10 series and plated 30 μL of each condition in one-quarter of a selective plate. Colonies were counted on the second day after plating.

## Results and Discussion

### Design of Postassembly Plasmid Amplification

**Figure 1:**
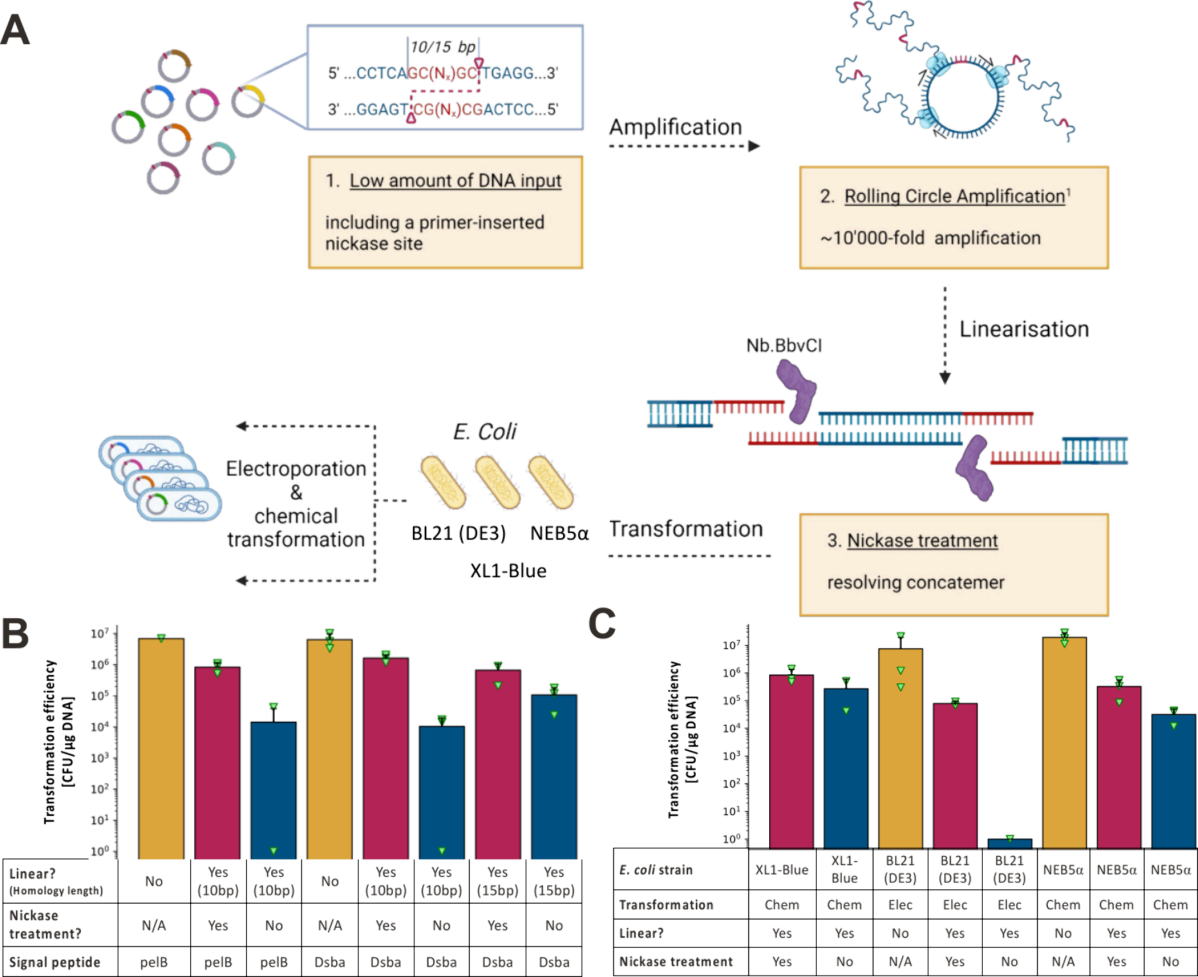
Testing the influence of sticky or blunt DNA overhangs while using supercoiled DNA as control for transformability of different *E. coli* cell strains. (A) Optimized post-assembly library amplification for transformation into a variety of *E. coli* strains (e.g., XL1--Blue, BL21 (DE3), and NEB5α). For simplicity, RCA is displayed without further branching of the first layer of elongated product. (B) Comparison of transformation efficiencies of the same *E. coli* strain (XL1-Blue) with the VHH-encoding plasmid (“PF-Nbb102-CAM”) across the different (1) formats: circular (orange), single-stranded homology site (red), double-stranded homology site (blue), (2) lengths of the homology sites (10 and 15 bp), and (3) signal-peptides (pelB and Dsba). Nonlinearized plasmids are in circular supercoiled format from plasmid extraction. (C) Assessing transformation efficiency of the same VHH plasmid (“PF-Nbb102-CAM”) in circular (orange) and linear formats across different *E. coli* strains (XL1-Blue, BL21 (DE3), and NEB5α). The linear format is further divided into plasmids with single-stranded homology sites (nickase-treated, red) and double-stranded homology sites (non-nickase treated, blue). Importantly, no rolling circle amplification was conducted prior to the experiment and all linear DNA products are from PCR amplification with appropriate primers to add the nickase sites. The abbreviations “Elec” and “Chem” in the row “transformation” stand for the transformation method “electroporation” and “chemical transformation” respectively, which are described in detail in the method section. All data are displayed as the mean of transformation triplicates (with individual data points displayed with green triangles). Error bars represent standard deviation of replicates and face only upward for simplicity.

Focusing on *E. coli* first, we designed our approach around three key techniques: 1) RCA of small-scale DNA assemblies to generate ∼100 μg of DNA, 2) nicking endonuclease treatment to resolve the RCA product into linear monomeric units, and 3) *in vivo* assembly of linear DNA by *E. coli* to generate circular plasmids (Figure 1A). Nicking endonuclease treatment was chosen based on observations from Xia et al.^9^ who reported that the generation of single-stranded homologous ends is sufficient to recapitulate the cloning and transformation efficiencies of the more complex Gibson assembly technique, and are more efficient than the transformation of linear DNA with double-stranded homologous ends seen in García-Nafría et al.^13^ Additionally, use of nicking endonucleases for cloning purposes has been successfully developed by Wang et al.^19^ Our test plasmid was thus modified to include a “nickase cassette”, in which two Nb. BbvCI sites were added on the top and bottom strand of the plasmid, respectively, such that upon nickase treatment a) the concatomeric RCA product will be resolved to monomers and b) the linear monomers will possess single-stranded 5′ and 3′ overhangs that are homologous to one another.

### Validation of Single-Strand Overhangs as Enhancers of Transformation Efficiency

As a first test of our approach, we investigated the effect of single-stranded homologous ends of DNA molecules on the transformation efficiency of *E. coli* across multiple homology lengths and *E. coli* strains. Initially, two different homology lengths (10 and 15 bases) were) added to the template DNA (a phagemid for VHH phage display, with either the pelB or DsbA signal peptides for periplasmic export of the VHH-p3 fusion protein) through PCR amplification. When subjecting the linear DNA products to nickase treatment, a substantial boost in transformation efficiency (10–100 times higher) was observed compared to the untreated PCR products (linear, no nickase treatment). Notably, the 10-base homology length resulted in a slightly superior transformation efficiency relative to the 15-base homology length in the context of the phagemid carrying the DsbA signal peptide (see Figure 1B). As such, we proceeded with the 10 base homology length and additionally tested the transformation efficiency of a pelB signal peptide containing phagemid in comparison to that of the DsbA containing phagemids. Little to no effect was seen; thus, we proceeded with the more standard pelB signal peptide in subsequent experiments. We then explored the transformation of different *E. coli* strains relevant to protein engineering (XL1-blue for phage display, BL21 (DE3) for protein expression, and NEB5α for cloning) using nickase-treated linear DNA, and observed the same effects as before, *i.e*., that single-stranded homologous ends enhanced transformation efficiency when compared to double-stranded homologous ends across all cell types (Figure 1C).

### Nickase-Mediated Resolution of Concatomeric RCA Products

**Figure 2:**
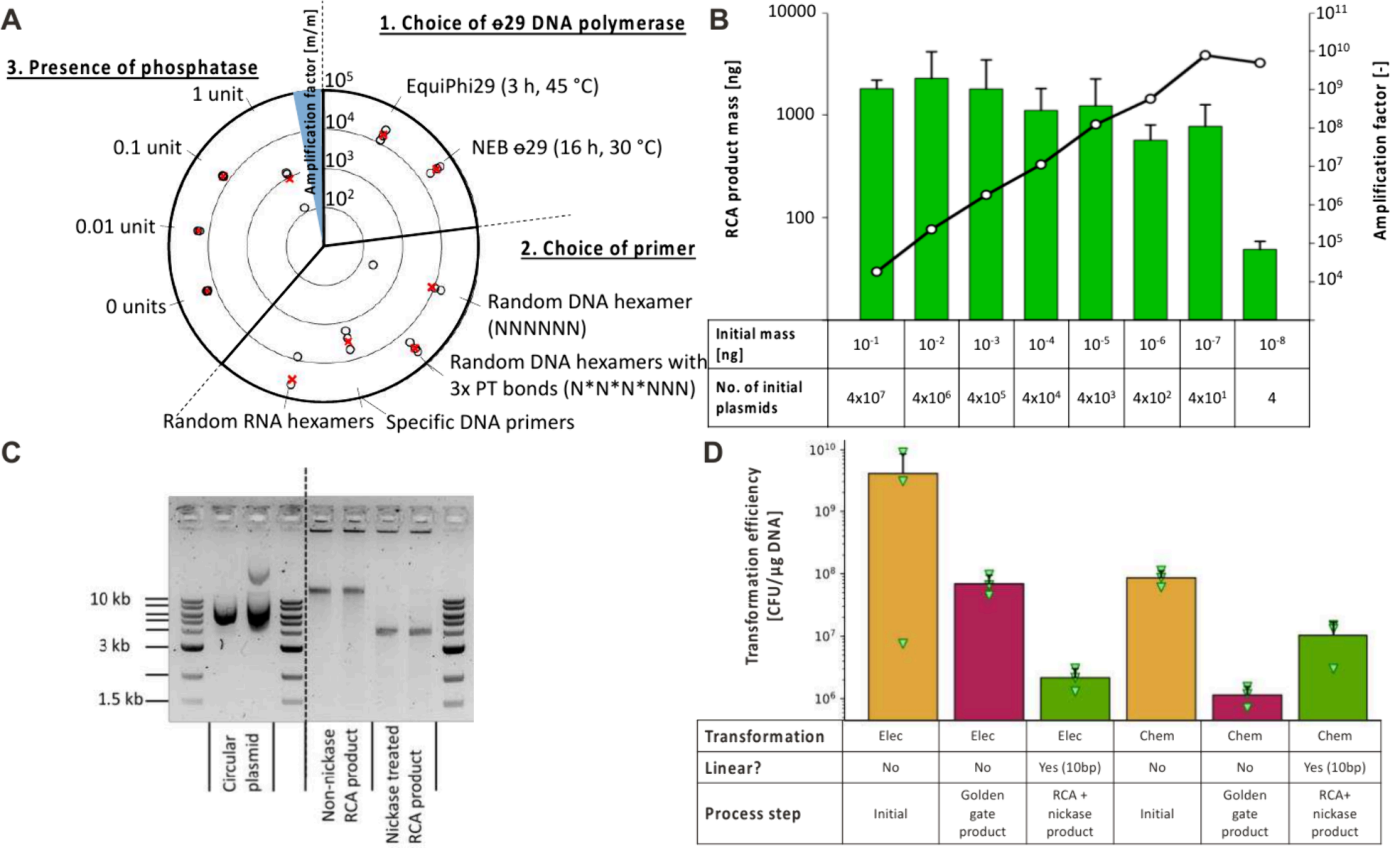
Robust and simplified library amplification using RCA. (A) Seeking to optimize conditions for RCA, several parameters were considered and evaluated when amplifying the plasmid “PF-Nbb102-CAM”. Going clockwise, first, (1) two ϕ29 polymerases (at 10 units per 20 μL reaction) were compared using 5 mM random hexamer primers, including three phosphorothioate (PT) bonds on the ′3 end, while following the suppliers’ recommendations regarding duration and temperature of the amplification step. Next (2), different primers (at 5 mM concentration) were assessed for their suitability. The specific DNA primer (more information is in the method section, as well as vector maps in the Supporting Information) was designed to one site of the selected plasmid. Furthermore, we tested whether the presence of phosphatase (NEB: M0262S) would benefit the amplification. Individual data points are shown as open circles (black), with their respective means (of triplicates) shown as crosses (red). (B) Comparing the output RCA product mass (left *y*-axis, green bars) to the input mass of plasmid DNA (labeled on the *x*-axis). Amplification factors (right *y*-axis, depicted with black/white dots connected by black line) versus input mass of plasmid DNA are also shown. Error bars reflect standard deviation over replicates of three for each condition. (C) Visualization of nickase-treated RCA product (lane 7 and 8 (left to right)) in comparison to the presumably supercoiled input plasmids (“PF-Nbb102-CAM” and “DF-Nbb102-CAM” in lanes 2 and 3, respectively) and the non-nickase treated RCA-products (lanes 5 and 6). The RCA reaction was performed using random DNA PT- and RNA hexamers (lanes 5/7 and 6/8, respectively). The RCA product trapped in the agarose gel pockets is likely high-molecular weight ssDNA which could, therefore, not be cut by Nb.BbvCI.^15^ The dashed line indicates that the image has been cropped for the sake of simplicity. The original version can be found in Figure S3. (D) Beyond establishing the parameters for DNA amplification and transformation into *E. coli*, we sought a better understanding of the transformation efficiencies (electroporation and chemical) over the *in vitro* process of library assembly. The plasmid “PF-Nbb102-CAM” was purified from an overnight culture and transformed either directly (conditions 1 and 4), or amplified by PCR for subsequent reinsertion of the VHH encoding sequence by Golden-Gate assembly. The purified Golden-Gate product was then either transformed directly (conditions 2 and 4) or subjected to RCA amplification and nickase treatment (using Nb.BbvCI) and subsequently transformed (conditions 3 and 6). Notably, all DNA samples were purified prior to the transformation. For the transformations purified plasmid (Electro: 13.9 ng, Chemical: 12.2 ng), Golden-Gate product (Electro: 20.3 ng, Chemical: 8.87 ng) or RCA amplified and nickase-treated dsDNA (Electro: 16.2 ng, Chemical: 10.5 ng) were applied to 50 μL of Electrocompetentent XL1-Blue *E. coli* cells (Titer: 4.8 × 10^10^ cells/ml) or 30 μL of chemically competent XL1-Blue cells (Titer: approximately 5.5 × 10^9^ cells/ml). Data are the mean of triplicates, with individual data points shown as triangles (green) and standard deviation shown by the capped line (upward facing only for simplicity).

After confirming that nickase treatment was beneficial, we investigated the best conditions for RCA of plasmids focusing on both amplification yield and specificity based on literature protocols.^20,21^ Importantly, the plasmid of interest now contained the 10 bp nickase cassette that resulted from transformation of the linear DNA products in Figure 1. This investigation uncovered that NEB Phi29 coupled with RNA or DNA (phosphorothioate-protected) random hexamers yielded the most product (>10,000 fold amplification) (Figure 2A). We also observed that the amplification yield increased linearly with reduced input template DNA concentration (Figure 2B), suggesting that a saturating quantity of product DNA is reached independent of input template concentration. Next, we sought to confirm that treatment with the nickase would resolve the concatomeric DNA product into monomeric units through agarose gel electrophoresis (Figure 2C). The RCA product seen beyond the upper limit (10 kb) of our ladder is resolved to the correct monomeric size of our plasmid, while the DNA trapped in the pocket of the gel is unaffected by nickase treatment likely due to it being ssDNA as also seen by Grasemann et al.^15^ We then investigated whether the RCA-nickase workflow could be simplified such that minimal buffer exchange or purification would be required. This approach identified the use of 1× NEB rCutSmart buffer with 0.1 mg/mL NEB rAlbumin as the most efficient, conveniently enabling both RCA and nickase treatment without purification or buffer exchange.

Upon confirmation of our workflow in the context of purified plasmid DNA, we next investigated the best conditions for the amplification of DNA in an *in vitro* assembly context, i.e., amplification of a Golden-Gate assembly. Amplification of purified, golden-gate assembled DNA was readily achieved with >18,000-fold amplification yield, generating 9.2 μg of DNA from 0.5 ng of input DNA (approximately 8.8 × 10^8^ DNA molecules) (Table 1). Post-nickase treatment and purification, a yield of ∼3 μg was achieved, representing an input to transformable output amplification yield of ∼6500 fold. In comparison, a similar PCR-based amplification workflow achieved an amplification yield ∼10-fold lower than the RCA workflow. The RCA workflow is readily scalable in terms of initial volume and output mass; e.g., parallelized (80×) amplification of 40.2 ng of Golden-Gate assembled product resulted in >700 μg of purified, nickase-treated plasmid target DNA. We noticed that treatment of the Golden-Gate product with exonuclease to remove unassembled insert DNA was not necessary (Figure S1).

**Table 1:**
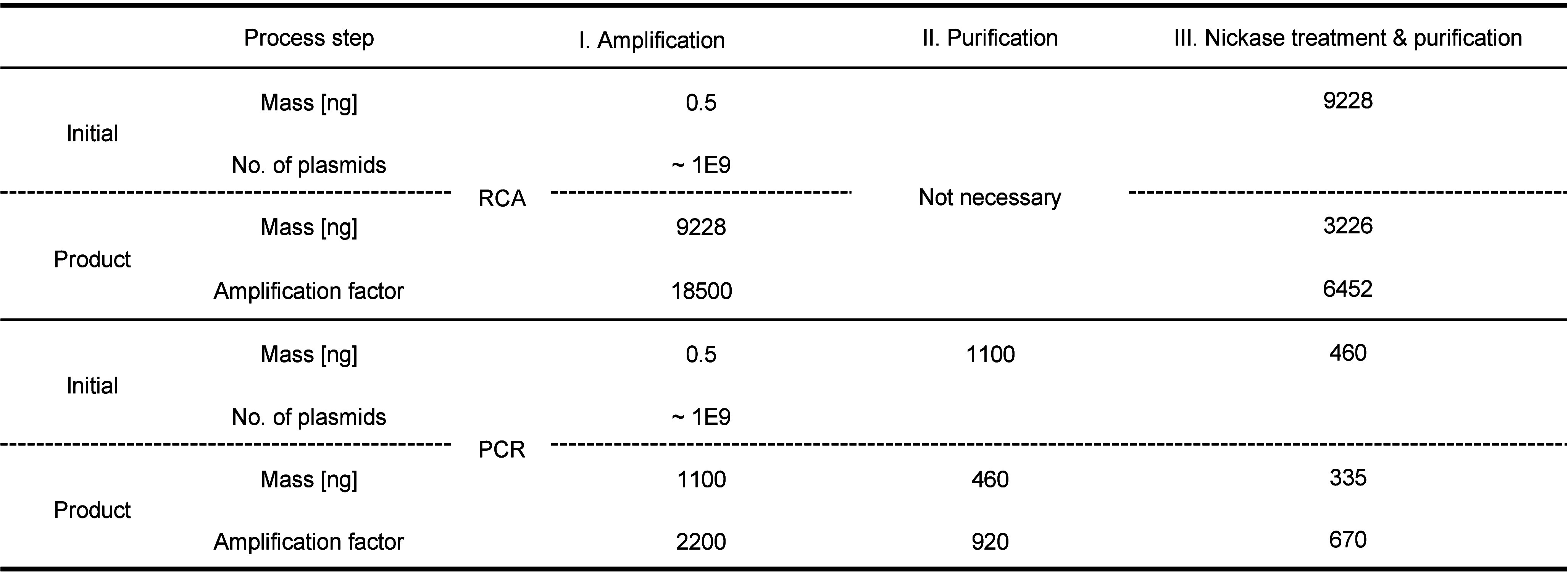
Quantification of DNA Library Amplification^a^

a Overview and comparison of the RCA and PCR amplification procedures by mass, total number of plasmids, and amplification factor. The values of mass are based on mass-over-volume concentration measurements, while the number of total plasmids and amplification factor are derived from the mass values and are, therefore, dependent on each other. The number of theoretical plasmids in the RCA input was estimated by predicting the mass of individual plasmid molecules is calculated from the molecular mass was executed using the web application NEBioCalculator (v1.15.4 May 23, 2023) and might not reflect natural isotopic abundances (Supporting Information).

We then looked at the transformation efficiencies of circular supercoiled plasmids, relaxed circular Golden-Gate assembled plasmids, and linear nickase-treated RCA product across both electroporation and chemical transformation of XL1-Blue *E. coli* cells (Figure 2D). As mentioned in the Introduction we observed the significant loss of transformation efficiency (10–100×) when comparing circular supercoiled DNA to the output of cloning reactions - a widely discussed but rarely quantified fact in the field. Additionally, we can approximate how many cells actually are transformed in the best case (circular supercoiled DNA): 2.3% for electrocompetent and 0.6% for chemically competent cells. Relevant to our nickase workflow, we observed that the chemically competent cells were transformed more efficiently (9×) with our linear nicked DNA than relaxed cloned circular DNA (Golden-Gate product), yet the opposite was true for electrocompetent cells (Golden-Gate product 31-fold greater than linear nicked DNA). Due to this observation, we explored a variety of parameters around chemical transformation, including cell type, transformation buffers, cell concentrations, storage media, growth media for competent cell production, and growth media for post-transformation selection plates, as well as the potential scalability of chemical transformations through parallelization, and the use of increased cell volumes (Figure S2). Most notably we observed that the TSS-HI^16^ protocol using strain BW25113 exhibited high variability of transformation efficiency when testing different plasmids (Figure S2A), the modified Inoue protocol compares favorably to the most efficient method using homemade Zymo Mix-and-Go cells (Figure S2B), plating cells on SOC agar significantly increases efficiency vs LB agar while both freezing storage media (DMSO or Glycerol) and cell up-concentrating has little effect (Figure S2C) and that parallel transformation in 96-well plates is as efficienct as in standard Eppendorf tubes (Figure S2D).

### RCA-Mediated Amplification of Plasmid DNA for *S. cerevisiae* Transformation

After establishing the use of the RCA nickase workflow for *E. coli*, we also tested its applicability to other common organisms used for biological experiments. We chose to investigate *S. cerevisiae*, notably the EBY100 strain for two reasons; first, EBY100 is commonly used for yeast surface display, a powerful technique for the ultrahigh throughput quantitative screening of binder libraries^23,24^, and second, because yeast is known to possess strong homologous recombination activity enabling transformation with linear DNA molecules, we expected that our homology-based cloning approach may have an effect on transformation efficiencies.

**Figure 3:**
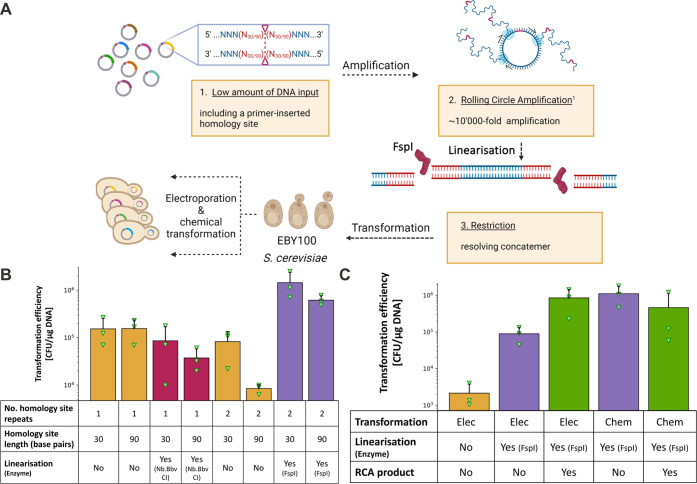
Testing DNA plasmid formats and modifications for optimal transformation into *S. cerevisiae* EBY100. (A) Schematic of optimized postassembly library amplification for transformation into *S. cerevisiae*. (B) Chemical transformation efficiencies in *S. cerevisiae* across different formats:circular (orange), linear single-stranded (red), and linear double-stranded (purple) and two different homology site lengths (30 and 90 base pairs) of similar plasmids (“pCT-antiGFP”). While the treatment of Nb.BbvCI resulted in a linear format with single-stranded 3′ overhangs, the double-stranded homology sites restricted by treatment with FspI leaves blunt ends. Vitally for the double-stranded homology, the according plasmid contains a repeat of the homology site, which enables *in vivo* processing and assembly. Notably, all DNA samples were purified prior to transformation. (C) Transformation of the RCA product (green) into chemical or electrocompetent cells. For chemical transformation linear FspI treated, non-RCA amplified (1.86 μg), and linear FspI treated, but RCA amplified plasmid (1.82 μg) were applied to 100 μL chemically competent EBY-100 cells at a titer of approximately 7.35 × 10^6^ cells/mL. Second, for the electroporation, linear FspI treated, non-RCA amplified (6 μg), linear FspI treated, but RCA amplified plasmid (7.68 μg) as well as circular, non-FspI and non-RCA amplified plasmid (7.65 μg) were applied to 400 μL of electrocompetent EBY-100 cells at a titer of approximately 8 × 10^7^ cells/mL. All data are the mean of triplicates, with individual data points shown as triangles (green) and standard deviation shown by the capped line (upward facing only for simplicity).

*S. cerevisiae* cells are often transformed with linear DNA possessing double-stranded homologous ends; thus, we created a new homology cassette in the yeast plasmids alongside our previously described nickase-single strand cassette. This new cassette contained an FspI site in the center of a 30 or 90 base repeat sequence, such that upon FspI treatment the RCA product would resolve to monomeric units with double-stranded homologous ends (Figure 3A). We observed that the linear FspI treated double-stranded homology DNA was most efficient in chemical transformation of yeast cells, outperforming both circular supercoiled DNA and the linear nickase treated single-stranded homology DNA by at least 10-fold (Figure 3B). This result established the utility of our approach in yeast, as well as providing data confirming that linear DNA transforms yeast at higher efficiency than circular DNA. As we had observed contradictory results in *E. coli* between electroporation and chemical transformation, we also tested electrocompetent yeast and observed that linearization significantly improved transformed efficiency versus circular DNA (Figure 3C).

## Conclusions

An increased yield of transformants from a defined initial quantity of *in vitro* assembled DNA is useful across many fields such as basic biology, sequencing, DNA storage, and directed evolution. Fields such as directed evolution rely upon the creation of large libraries of transformants from cloned DNA, and can significantly benefit from streamlined processes to achieve this. Novel technologies, such as *in vivo* hypermutation (e.g., in *E. coli* as PACE^25^/PRANCE^26^ or MutaT7,^27^*Bacillus thuringiensis* as BacORep,^28^ and *S. cerevisiae* as OrthoRep^29^), can circumvent the need to physically transform as many cells as you want your library size to comprise. Yet it is still likely that physical transformation will remain the method of choice for many researchers due to specific library designs, or the choice of screening technologies which are not compatible with the mentioned technologies.

In this work, we have developed a simple protocol for increasing the yield of transformants from an initial amount of *in vitro* assembled DNA, through post-assembly amplification and linearization to appropriate formats for transformation into both *E. coli* and *S. cerevisiae*. While it was an unintended consequence of the interesting molecular biology that we uncovered that linear DNA does not transform efficiently via electroporation, the outcome of focusing on chemical transformation into *E. coli* is elegant as it requires access to fewer specific pieces of equipment. The observed difference in efficiency between linear DNA transformation into electrocompetent cells and chemically competent cells can likely be explained by the unique mechanisms by which DNA passes into the cells of each approach. However, it is difficult to expand much beyond this hypothesis as the exact mechanisms of either chemical competency or electrocompetency are not fully understood. Throughout, we sought to optimize our protocols for robustness, simplicity, and scalabilty and thus explored many criteria not often quantified. For instance, the effect of the media used to plate transformations on and the discrepancy between efficiencies seen with circular supercoiled DNA (as commercial cells are often advertised using) and DNA in a library context (i.e., *in vitro* assembled).

To further extend the utility of our approach, we confirmed its use for increasing transformant yields in *S. cerevisiae*, a highly useful organism in biotechnology and synthetic biology for which RCA-mediated DNA amplification has not been explored previously. We expect that the product of our protocol could be of benefit in organisms other than *E. coli* or *S. cerevisiae*, such as the rapidly developing biotechnology chassis organism *Vibrio natriegens*(30) for which efficient and scalable natural transformation techniques have been established.^31^ Additionally, the linear DNA product of our protocol could be used for genome integration rather than plasmid formation for a wide variety of applicable organisms.

In conclusion, our workflow can readily yield ∼6500- fold amplification of *in vitro* assembled DNA in a format suited to efficient transformation into either *E. coli* or *S. cerevisiae* yielding more transformants from a defined amount of starting DNA. This is achieved in the simplest way possible, with a focus on reduction of both cost and resource usage such that more people can access powerful approaches such as directed evolution or genome engineering.

## Data Availability

All underlying raw data files and calculations are accessible.

## Supporting Information Available

Effect of exonuclease treatment in the process of library amplification using RCA on subsequent transformation efficiency, benchmarking chemical transformation methods, full image of RCA concatomer resolution gel, sequences of plasmids used in this study (PDF)

## Abbreviations

DsbA: signalpeptide leader sequence of DsbA in *Escherichia coli*
pelB: signalpeptide leader sequence of pectatelyase B of *Erwinia carotovora*
RCA: rolling circle amplification
SOB: super optimal broth
SOC: super optimal broth with catabolite repression
VHH: heavy-chain variable domain
V. natriegens: *Vibirio natriegens*
