# Supplementary material for: Post-assembly Plasmid Amplification for Increased Transformation Yields in E. coli and S. cerevisiae
Source: Chem Bio Eng. 2024 Nov 18;2(2):87–96. doi: 10.1021/cbe.4c00115 (PMC11873849; doi:10.1021/cbe.4c00115)
Supplement: Supplementary file 1 — be4c00115_si_001.pdf [file be4c00115_si_001.pdf]

**SUPPORTING INFORMATION**

Post-Assembly Plasmid Amplification for Increased Transformation Yields in *E. coli* and *S. cerevisiae*

Thomas Fryer[1,2†], Darian S. Wolff[1,2†], Max D. Overath[1], Elena Schäfer[3], Andreas H. Laustsen[1*], Timothy P. Jenkins[1*], Carsten Andersen[2*]

1 Department of Biotechnology and Biomedicine, Technical University of Denmark, Søltofts Plads 239, Lyngby, Hovedstaden, DK 2800, Denmark

2 Department of Molecular Discovery, R&D, Novozymes A/S, Bagsvaerd, Hovedstaden, DK 2880, Denmark

3 Department of Biochemistry, University of Cambridge, CB2 1GA, United Kingdom

* Corresponding authors: ahola@bio.dtu.dk, cara@novozymes.com, tpaje@dtu.dk

† Equal contribution

Supporting figures and tables:

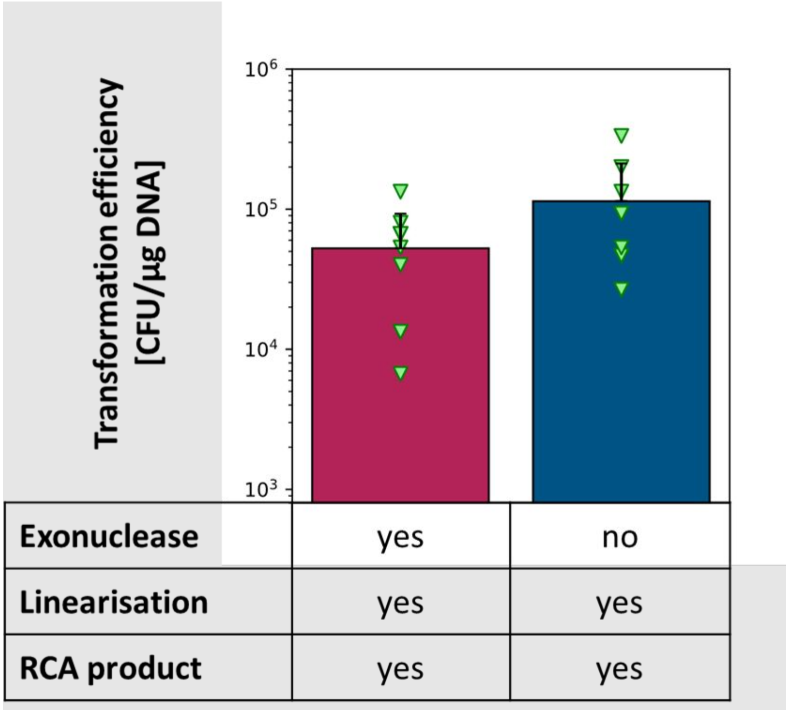


**Figure S1 Effect of exonuclease treatment in the process of library amplification using RCA on subsequent transformation efficiency.** To further optimise the library creation process, we assessed whether treatment with exonuclease V (NEB, Catalogue-no.: M0345L), a RecBCD complex from *E. coli*, would have an effect on transformation efficiency. Exonuclease V bidirectionally hydrolyses phosphodiester bonds between nucleotides in linear double-stranded DNA, leaving ideally only assembled DNA after golden-gate assembly of the VHH-containing insert into a suitable vector (‘Pf-Nb-b102-Cam-lin10’). From the calculated transformation efficiency of the individual samples and their replicates (depicted in green triangles), average (highlighted by red (including exonuclease treatment) and blue bars), and standard deviation were noted. Following the manufacturer’s instructions, 10 units of exonuclease V were employed per 1 µg of DNA, while adding 1 mM ATP. Reaction was quenched after 30 min by adding >10 mM EDTA. Error bars represent standard deviation of replicates and face only upwards for simplicity. Individual replicates are highlighted in green triangles.

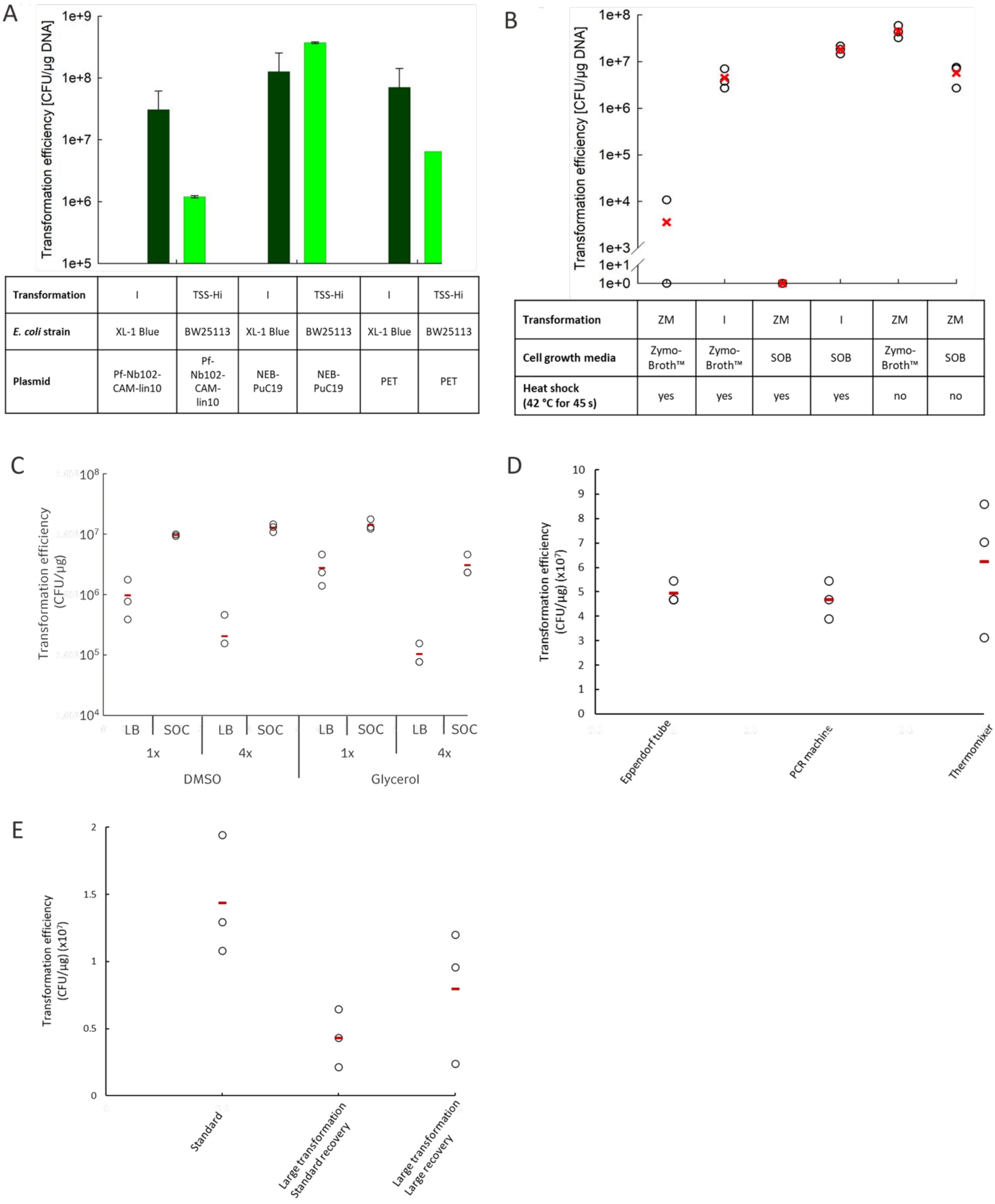


| Transformation | I | TSS-Hi | I | TSS-Hi | I | TSS-Hi |
|---|---|---|---|---|---|---|
| *E. coli* strain | XL-1 Blue | BW25113 | XL-1 Blue | BW25113 | XL-1 Blue | BW25113 |
| Plasmid | Pf-Nb102-CAM-lin10 | Pf-Nb102-CAM-lin10 | NEB-PuC19 | NEB-PuC19 | PET | PET |

| Transformation | ZM | I | ZM | I | ZM | ZM |
|---|---|---|---|---|---|---|
| Cell growth media | Zymo-Broth™ | Zymo-Broth™ | SOB | SOB | Zymo-Broth™ | SOB |
| Heat shock (42 °C for 45 s) | yes | yes | yes | yes | no | no |

**Figure S2. Benchmarking chemical transformation methods. (A)** Seeking the best performance, the current method of chemical *E. coli* transformation, named ‘I’ referring to ‘Inoue’ in dark green, was evaluated in transformation efficiency over the range of three different plasmids against the ‘TSS-HI’ method depicted in light green. (**B**) Next, ‘I’ was tested against the commercial *E. coli* transformation kit Zymo Mix&Go (Zymo Research, catalogue-no.: T3001). Most noticeably, a heat shock procedure

(heating to 42 °C for 45 sec. and immediate cooling on ice) is not included in the protocol of Zymo research. We, therefore, tried different combinations of transformation protocol, media, and heat shock. (**C**) Chemically competent XL-1 Blue cells were prepared as per the Inoue protocol, with differences in their final storage formulation (DMSO or glycerol), how concentrated the aliquots were (with 4x denoting a resuspension in a 4-fold lower final volume), and what agar the transformations were plated on (LB Cam, or SOC 2% Glucose Cam). (**D**) Parallelisation of transformations was explored by comparing standard protocols (in an eppendorf tube) to use of a 96-well PCR plate containing aliquots of cells and heat-shock in a PCR machine (programmed to 4 $^{o}$C for 30 minutes, 42 $^{o}$C for 45 seconds, and 4 $^{o}$C for 5 minutes) or in 96-well metallic cold blocks on ice and a 96-well thermomixer programmed to 42 $^{o}$C (**E**) The volumetric scale-up of transformation was explored by comparing standard transformations in Eppendorf tubes (100 µL cells + 900 µL recovery media) to transformation of 1 mL cells in an Eppendorf tube (large transformation) and either standard recovery (100 µL cells + 900 µL recovery media) or large recovery (900 µL cells + 9000 µL recovery media). Data are the mean of triplicates (red) with individual data points indicated.

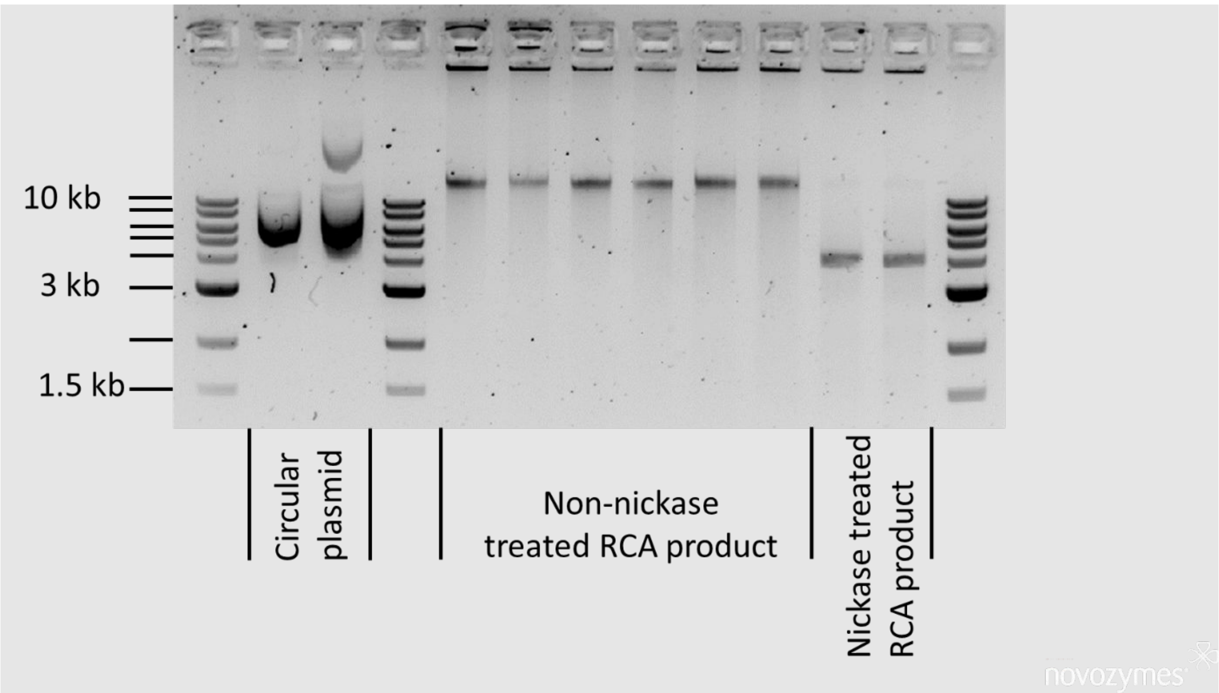


**Figure S3. Nickase-mediated resolution of concatermeric, RCA amplified plasmid.** Please note that this is the same image as in Figure 2C, but not cropped. Visualisation of nickase-treated RCA product (lane 11 and 12 (left to right)) in comparison to the presumably supercoiled input plasmid ('PF-Nbb102-CAM' and 'DF-Nbb102-CAM' in lane 2, respective 3) and the non-nickase treated linear RCA-products (lanes 5-10). The RCA reaction was performed using random DNA PT- and RNA hexamers (lane 5, 7, 9, and 11, and 6, 8, 10, and 12, respectively). Furthermore, to obtain an accurate comparison to the nickase-treated samples, the RCA product in lane 9 and 10 underwent the same treatment of buffers, heat, and additional purification without addition of the nickase enzyme Nb.BbvCI as the nickase treated samples in lane 11 and 12. Samples in lane 5 and 6 were not purified after the RCA reaction, while the samples in lane 7 to 10 were purified using SPRI beads.

**Table S1: Plasmid sequences**

| Plasmid | Sequence 5’-3’ (relevant cassette region in bold and uppercase, with restriction site(s) underlined) |
|---|---|
| pCT-antiGFP Nick 30 | ccaatacgcaaaccgcctctccccgcgcgttggccgattcattaatgcagctggcacgacaggtttcccgactggaaagcgggcagtgagcgcaacgcaatt<br>aatgtgagttacctcactcattaggcaccccaggctttacactttatgcttccggctcctatgttgtgtggaattgtgagcggataacaatttcacacaggaaacag<br>ctatgaccatgattacgccaagctgccagatctgcagccgctatatatccgcggattaacgcgctagccggctgggccgtcgaacggaattaaccctcactaa<br>agggaacaaaagctgggtacccgacaggttatcagcaacaacacagtcatatccattctcaattagctctaccacagtgtgtgaaccaatgtatccagcacca<br>cctgtaaccaaaacaattttagaagtactttcactttgtaactgagctgtcatttatattgaattttcaaaaattcttactttttttttggatggacgcaaagaagtttaata<br>atcatattacatggcattaccaccatatacatatccatatacatatccatatctaatcttacttatatgttgtggaaatgtaaagagccccattatcttagcctaaaaaa<br>accttctctttggaactttcagtaatacgcttaactgctcattgctatattgaagtacggattagaagccgccgagcgggtgacagccctccgaaggaagactctc<br>ctccgtgcgtcctcgtcttcaccggtcgcgttcctgaaacgcagatgtgcctcgcgccgcactgctccgaacaataaagattctacaatactagcttttatggttatg<br>aagaggaaaaattggcagtaacctggccccacaaaccttcaaatgaacgaatcaaattaacaaccataggatgataatgcgattagttttttagccttatttctg<br>gggtaattaatcagcgaagcgatgatttttgatctattaacagatatataaatgcaaaaactgcataaccactttaactaatactttcaacattttcggtttgtattactt<br>cttattcaaatgtaataaaagtatcaacaaaaaattgttaatatacctctatactttaacgtcaaggagaaaaaaccccggatcgaattccctacttcatacattttc<br>aattaagatgcagttacttcgctgtttttcaatattttctgttattgcttcagttttagcacaggaactgacaactatatgcgagcaaatcccctcaccaactttagaatc<br>gacgccgtactctttgtcaacgactactattttggccaacgggaaggcaatgcaaggagtttttgaatattacaaatcagtaacgtttgtcagtaattgcggttctca<br>cccctcaacaactagcaaaggcagccccataaacacacagtatgtttttaaggacaatagctcgacgattgaaggtagatacccatacgacgttccagacta<br>cgctctgcaggctagtggtggcggaggttctggtggaggcgggtctggtgggggaggatctgccatggtgcagctggtggaaagcggtggtgcactggtgca<br>gccaggtggcagcctgcgtctgagctgcgcggcgagcggctttccggtgaaccgctatagcatgcgctggtatcgtcaggcgccgggcaaagaacgcgaat<br>gggtggcgggcatgagcagcgcgggcgatcgcagcagctatgaagatagcgtgaaaggccgctttaccattagccgcgatgatgcgcgcaacaccgtgta<br>tctgcagatgaacagcctgaaaccggaagataccgcggtgtattattgcaacgtgaacgtgggctttgaatattggggccagggcacccaggtgaccgtgag<br>cagcgcggccgctagcgaaaccccaggtacgagcgaaagcgcgaccccggaacatatggtgccgaccatcgtgatggtggacgcctacaagcgttaca<br>agtaatagctcgagatctgataacaacagtgtagatgtaacaaaatcgactttgttcccactgtacttttagctcgtacaaaatacaatatacttttcatttctccgtaa<br>acaacatgttttcccatgtaatatccttttctatttttcgttccgttaccaactttacacatactttatatagctattcacttctatacactaaaaaactaagacaattttaattt<br>tgctgcctgccatatttcaatttgttataaattcctataatttatcctattagtagctaaaaaaagatgaatgtgaatcgaatcctaagagaattgag**CCTCAGC**<br>**AGGTGGCCGATTCATTAATGCAGTTTGCTGAGG**ctccaattcgccctatagtgagtcgtattacaattcactggccgtcgttttacaacgtc<br>gtgactgggaaaaccctggcgttacccaacttaatcgccttgcagcacatccccctttcgccagctggcgtaatagcgaagaggcccgcaccgatcgccctttcc<br>caacagttgggcagcctgaatggcgaatggacgcgccctgtagcggcgcattaagcgcggcgggtgtggtggttacgcgcagcgtgaccgctacacttgcc<br>agcgccctagcgcccgctcctttcgctttcttcccttcctttctcgccacgttcgccggctttccccgtcaagctctaaatcgggggctccctttagggttccgatttagt<br>gctttacggcacctcgaccccaaaaaacttgattagggtgatggttcacgtagtgggccatcgccctgatagacggtttttcgccctttgacgttggagtccacgtt<br>ctttaatagtggactcttgttccaaactggaacaacactcaaccctatctcggtctattcttttgatttataagggattttgccgatttcggcctattggttaaaaaatgag<br>ctgatttaacaaaaatttaacgcgaattttaacaaaatattaacgcttacaatttcctgatgcggtattttctccttacgcatctgtgcggtatttcacaccgcatagatc<br>ggcaagtgcacaaacaatacttaaataaatactactcagtaataacctatttcttagcatttttgacgaaatttgctattttgttagagtcttttacaccatttgtctccac<br>acctccgcttacatcaacaccaataacgccatttaatctaagcgcatcaccaacattttctggcgtcagtccaccagctaacataaaatgtaagctttcggggctc<br>tcttgccttccaacccagtcagaaatcgagttccaatccaaaagttcacctgtcccacctgcttctgaatcaaacaagggaataaacgaatgaggtttctgtgaa<br>gctgcactgagtagtatgttgcagtcttttggaaatacgagtcttttaataactggcaaaccgaggaactcttggtattcttgccacgactcatctccatgcagttgga<br>cgatatcaatgccgtaatcattgaccagagccaaaacatcctccttaggttgattacgaaacacgccaaccaagtatttcggagtgcctgaactatttttatatgct<br>tttacaagacttgaaattttccttgcaataaccgggtcaattgttctctttctattgggcacacatataatacccagcaagtcagcatcggaatctagagcacattctg<br>cggcctctgtgctctgcaagccgcaaactttcaccaatggaccagaactacctgtgaaattaataacagacatactccaagctgcctttgtgtgcttaatcacgta<br>tactcacgtgctcaatagtcaccaatgccctccctcttggccctctccttttcttttttcgaccgaattaattcttaatcggcaaaaaaagaaaagctccggatcaaga<br>ttgtacgtaaggtgacaagctatttttcaataaagaatatcttccactactgccatctggcgtcataactgcaaagtacacatatattacgatgctgtctattaaatgct<br>tcctatattatatatatagtaatgtcgtttatggtgcactctcagtacaatctgctctgatgccgcatagttaagccagccccgacacccgccaacacccgctgacg<br>cgccctgacgggcttgtctgctcccggcatccgcttacagacaagctgtgaccgtctccgggagctgcatgtgtcagaggttttcaccgtcatcaccgaaacgc<br>gcgagacgaaagggcctcgtgatacgcctatttttataggttaatgtcatgataataatggtttcttaggacggatcgcttgcctgtaacttacacgcgcctcgtatct |

| | |
|---|---|
| | tttaatgatggaataatttgggaatttactctgtgtttatttatttttatgttttgtatttggattttagaaagtaaataaagaaggtagaagagttacggaatgaagaaaa<br>aaaaataaacaaaggtttaaaaaatttcaacaaaaagcgtactttacatatatatttattagacaagaaaagcagattaaatagatatacattcgattaacgata<br>agtaaaatgtaaaatcacaggattttcgtgtgtggtcttctacacagacaagatgaaacaattcggcattaatacctgagagcaggaagagcaagataaaag<br>gtagtatttgttggcgatccccctagagtcttttacatcttcggaaaacaaaaactatttttttctttaatttcttttttttactttctatttttaatttatatatttatattaaaaaattta<br>aattataattatttttatagcacgtgatgaaaaggacccaggtggcacttttcggggaaatgtgcgcggaacccctatttgtttatttttctaaatacattcaaatatgta<br>tccgctcatgtcgagacgttgggtgaggttccaactttcaccataatgaaataagatcactaccgggcgtattttttgagttatcgagattttcaggagctaaggaa<br>gctaaaatggagaaaaaaatcactggatataccaccgttgatatatcccaatggcatcgtaaagaacattttgaggcatttcagtcagttgctcaatgtacctata<br>accagaccgttcagctggatattacggcctttttaaagaccgtaaagaaaaataagcacaagttttatccggcctttattcacattcttgcccgcctgatgaatgctc<br>acccggagttccgtatggcaatgaaagacggtgagctggtgatatgggatagtgttcacccttgttacaccgttttccatgagcaaactgaaacgttttcatcgctc<br>tggagtgaataccacgacgatttccggcagtttctacacatatattcgcaagatgtggcgtgttacggtgaaaacctggcctatttccctaaagggtttattgagaa<br>tatgtttttcgtctcagccaatccctgggtgagtttcaccagttttgatttaaacgtggctaatatggacaacttcttcgcccccgttttcaccatgggcaaatattatacg<br>caaggcgacaaggtgctgatgccgctggcgattcaggttcatcatgccgtttgtgatggcttccatgtcggcagaatgcttaatgaattacaacagtactgcgatg<br>agtggcagggcggggcgtaattttttttaaggcagttattggtgcccttaaacgcctggtgctacgcctgaataagtgataataagcggatgaatggcagaaattc<br>gaaagcaaattcgacccggtcgtcggttcagggcagggtcgttaaatagccgcttatgtctattgctggtttaccggtttattgactaccggaagcagtgtgaccgt<br>gtgcttctcaaatgcctgaggccagtttgctcaggctctccccgtggaggtaataattgctcgacatgaccaaaatcccttaacgtgagttttcgttccactgagcgt<br>cagaccccgtagaaaagatcaaaggatcttcttgagatcctttttttctgcgcgtaatctgctgcttgcaaacaaaaaaaccaccgctaccagcggtggtttgtttg<br>ccggatcaagagctaccaactctttttccgaaggtaactggcttcagcagagcgcagataccaaatactgtccttctagtgtagccgtagttaggccaccacttc<br>aagaactctgtagcaccgcctacatacctcgctctgctaatcctgttaccagtggctgctgccagtggcgataagtcgtgtcttaccgggttggactcaagacgat<br>agttaccggataaggcgcagcggtcgggctgaacggggggttcgtgcacacagcccagcttggagcgaacgacctacaccgaactgagatacctacagc<br>gtgagcattgagaaagcgccacgcttcccgaagggagaaaggcggacaggtatccggtaagcggcagggtcggaacaggagagcgcacgagggagc<br>ttccagggggaaacgcctggtatctttatagtcctgtcgggtttcgccacctctgacttgagcgtcgatttttgtgatgctcgtcaggggggccgagcctatggaaa<br>aacgccagcaacgcggcctttttacggttcctggccttttgctggccttttgctcacatgttctttcctgcgttatcccctgattctgtggataaccgtattaccgcctttga<br>gtgagctgataccgctcgccgcagccgaacgaccgagcgcagcgagtcagtgagcgaggaagcggaagagcgc |
| pCT anti-GFP Nick 90 | ccaatacgcaaaccgcctctccccgcgcgttggccgattcattaatgcagctggcacgacaggtttcccgactggaaagcgggcagtgagcgcaacgcaatt<br>aatgtgagttacctcactcattaggcaccccaggctttacactttatgcttccggctcctatgttgtgtggaattgtgagcggataacaatttcacacaggaaacag<br>ctatgaccatgattacgccaagctgccagatctgcagccgctatatatccgcggattaacgcgctagccggctgggccgtcgaacggaattaaccctcactaa<br>agggaacaaaagctgggtacccgacaggttatcagcaacaacacagtcatatccattctcaattagctctaccacagtgtgtgaaccaatgtatccagcacca<br>cctgtaaccaaaacaattttagaagtactttcactttgtaactgagctgtcatttatattgaattttcaaaaattcttactttttttttggatggacgcaaagaagtttaata<br>atcatattacatggcattaccaccatatacatatccatatacatatccatatctaatcttacttatatgttgtggaaatgtaaagagccccattatcttagcctaaaaaa<br>accttctctttggaactttcagtaatacgcttaactgctcattgctatattgaagtacggattagaagccgccgagcgggtgacagccctccgaaggaagactctc<br>ctccgtgcgtcctcgtcttcaccggtcgcgttcctgaaacgcagatgtgcctcgcgccgcactgctccgaacaataaagattctacaatactagcttttatggttatg<br>aagaggaaaaattggcagtaacctggccccacaaaccttcaaatgaacgaatcaaattaacaaccataggatgataatgcgattagttttttagccttatttctg<br>gggtaattaatcagcgaagcgatgatttttgatctattaacagatatataaatgcaaaaactgcataaccactttaactaatactttcaacattttcggtttgtattactt<br>cttattcaaatgtaataaaagtatcaacaaaaaattgttaatatacctctatactttaacgtcaaggagaaaaaaccccggatcgaattccctacttcatacattttc<br>aattaagatgcagttacttcgctgtttttcaatattttctgttattgcttcagttttagcacaggaactgacaactatatgcgagcaaatcccctcaccaactttagaatc<br>gacgccgtactctttgtcaacgactactattttggccaacgggaaggcaatgcaaggagtttttgaatattacaaatcagtaacgtttgtcagtaattgcggttctca<br>cccctcaacaactagcaaaggcagccccataaacacacagtatgtttttaaggacaatagctcgacgattgaaggtagatacccatacgacgttccagacta<br>cgctctgcaggctagtggtggcggaggttctggtggaggcgggtctggtgggggaggatctgccatggtgcagctggtggaaagcggtggtgcactggtgca<br>gccaggtggcagcctgcgtctgagctgcgcggcgagcggctttccggtgaaccgctatagcatgcgctggtatcgtcaggcgccgggcaaagaacgcgaat<br>gggtggcgggcatgagcagcgcgggcgatcgcagcagctatgaagatagcgtgaaaggccgctttaccattagccgcgatgatgcgcgcaacaccgtgta<br>tctgcagatgaacagcctgaaaccggaagataccgcggtgtattattgcaacgtgaacgtgggctttgaatattggggccagggcacccaggtgaccgtgag<br>cagcgcggccgctagcgaaaccccaggtacgagcgaaagcgcgaccccggaacatatggtgccgaccatcgtgatggtggacgcctacaagcgttaca<br>agtaatagctcgagatctgataacaacagtgtagatgtaacaaaatcgactttgttcccactgtacttttagctcgtacaaaatacaatatacttttcatttctccgtaa<br>acaacatgttttcccatgtaatatccttttctatttttcgttccgttaccaactttacacatactttatatagctattcacttctatacactaaaaaactaagacaattttaattt |

| | |
|---|---|
| | tgctgcctgccatatttcaatttgttataaattcctataatttatcctattagtagctaaaaaaagatgaatgtgaatcgaatcctaagagaattgag**CCTCAGCAGGGGCATCCCTCCTTTCAAGATAAATAATTTATACACTATTCTATTGGAATCTTAATCATTCTGGCCGATTCATTAATGCAGTTTGCTGAGG**ctccaattcgccctatagtgagtcgtattacaattcactggccgtcgttttacaacgtcgtgactgggaaaaccctggcgttacccaacttaatcgccttgcagcacatccccctttcgccagctggcgtaatagcgaagaggcccgcaccgatcgcccttcccaacagttgcgcagcctgaatggcgaatggacgcgccctgtagcggcgcattaagcgcggcgggtgtggtggttacgcgcagcgtgaccgctacacttgccagcgccctagcgcccgctcctttcgctttcttcccttcctttctcgccacgttcgccggctttccccgtcaagctctaaatcgggggctccctttagggttccgatttagtgctttacggcacctcgaccccaaaaaacttgattagggtgatggttcacgtagtgggccatcgccctgatagacggtttttcgccctttgacgttggagtccacgttctttaatagtggactcttgttccaaactggaacaacactcaaccctatctcggtctattcttttgatttataagggattttgccgatttcggcctattggttaaaaaatgagctgatttaacaaaaatttaacgcgaattttaacaaaatattaacgcttacaatttcctgatgcggtattttctccttacgcatctgtgcggtatttcacaccgcatagatcggcaagtgcacaaacaatacttaaataaatactactcagtaataacctatttcttagcatttttgacgaaatttgctattttgttagagtcttttacaccatttgtctccacacctccgcttacatcaacaccaataacgccatttaatctaagcgcatcaccaacattttctggcgtcagtccaccagctaacataaaatgtaagctttcggggctctcttgccttccaacccagtcagaaatcgagttccaatccaaaagttcacctgtcccacctgcttctgaatcaaacaagggaataaacgaatgaggtttctgtgaagctgcactgagtagtatgttgcagtcttttggaaatacgagtcttttaataactggcaaaccgaggaactcttggtattcttgccacgactcatctccatgcagttggacgatatcaatgccgtaatcattgaccagagccaaaacatcctccttaggttgattacgaaacacgccaaccaagtatttcggagtgcctgaactatttttatatgcttttacaagacttgaaattttccttgcaataaccgggtcaattgttctctttctattgggcacacatataatacccagcaagtcagcatcggaatctagagcacattctgcggcctctgtgctctgcaagccgcaaactttcaccaatggaccagaactacctgtgaaattaataacagacatactccaagctgcctttgtgtgcttaatcacgtatactcacgtgctcaatagtcaccaatgccctccctcttggccctctcctttttcttttttcgaccgaattaattcttaatcggcaaaaaaagaaaagctccggatcaagattgtacgtaaggtgacaagctatttttcaataaagaatatcttccactactgccatctggcgtcataactgcaaagtacacatatattacgatgctgtctattaaatgcttcctatattatatatatagtaatgtcgtttatggtgcactctcagtacaatctgctctgatgccgcatagttaagccagccccgacacccgccaacacccgctgacgcgccctgacgggcttgtctgctcccggcatccgcttacagacaagctgtgaccgtctccgggagctgcatgtgtcagaggttttcaccgtcatcaccgaaacgcgcgagacgaaagggcctcgtgatacgcctatttttataggttaatgtcatgataataatggtttcttaggacggatcgcttgcctgtaacttacacgcgcctcgtatcttttaatgatggaataatttgggaatttactctgtgtttatttatttttatgttttgtatttggattttagaaagtaaataaagaaggtagaagagttacggaatgaagaaaaaaaaataaacaaaggtttaaaaaatttcaacaaaaagcgtactttacatatatatttattagacaagaaaagcagattaaatagatatacattcgattaacgataagtaaaatgtaaaatcacaggattttcgtgtgtggtcttctacacagacaagatgaaacaattcggcattaatacctgagagcaggaagagcaagataaaaggtagtatttgttggcgatccccctagagtcttttacatcttcggaaaacaaaaactattttttcttaatttctttttttactttctatttttaatttatatatttatattaaaaaatttaaattataattatttttatagcacgtgatgaaaaggacccaggtggcacttttcggggaaatgtgcgcggaacccctatttgtttatttttctaaatacattcaaatatgtatccgctcatgtcgagacgttgggtgaggttccaactttcaccataatgaaataagatcactaccgggcgtattttttgagttatcgagattttcaggagctaaggaagctaaaatggagaaaaaaatcactggatataccaccgttgatatatcccaatggcatcgtaaagaacattttgaggcatttcagtcagttgctcaatgtacctataaccagaccgttcagctggatattacggcctttttaaagaccgtaaagaaaaataagcacaagttttatccggcctttattcacattcttgcccgcctgatgaatgctcacccggagttccgtatggcaatgaaagacggtgagctggtgatatgggatagtgttcacccttgttacaccgttttccatgagcaaactgaaacgttttcatcgctctggagtgaataccacgacgatttccggcagtttctacacatatattcgcaagatgtggcgtgttacggtgaaaacctggcctatttccctaaagggtttattgagaatatgtttttcgtctcagccaatccctgggtgagtttcaccagttttgatttaaacgtggctaatatggacaacttcttcgcccccgttttcacgatgggcaaatattatacgcaaggcgacaaggtgctgatgccgctggcgattcaggttcatcatgccgtttgtgatggcttccatgtcggcagaatgcttaatgaattacaacagtactgcgatgagtggcagggcggggcgtaattttttttaaggcagttattggtgcccttaaacgcctggtgctacgcctgaataagtgataataagcggatgaatggcagaaattcgaaagcaaattcgacccggtcgtcggttcagggcagggtcgttaaatagccgcttatgtctattgctggtttaccggtttattgactaccggaagcagtgtgaccgtgtgcttctcaaatgcctgaggccagtttgctcaggctctccccgtggaggtaataattgctcgacatgaccaaaatcccttaacgtgagttttcgttccactgagcgtcagaccccgtagaaaagatcaaaggatcttcttgagatcctttttttctgcgcgtaatctgctgcttgcaaacaaaaaaaccaccgctaccagcggtggtttgtttgccggatcaagagctaccaactctttttccgaaggtaactggcttcagcagagcgcagataccaaatactgtccttctagtgtagccgtagttaggccaccacttcaagaactctgtagcaccgcctacatacctcgctctgctaatcctgttaccagtggctgctgccagtggcgataagtcgtgtcttaccgggttggactcaagacgatagttaccggataaggcgcagcggtcgggctgaacggggggttcgtgcacacagcccagcttggagcgaacgacctacaccgaactgagatacctacagcgtgagcattgagaaagcgccacgcttcccgaagggagaaaggcggacaggtatccggtaagcggcagggtcggaacaggagagcgcacgagggagcttccagggggaaacgcctggtatctttatagtcctgtcgggtttcgccacctctgacttgagcgtcgatttttgtgatgctcgtcaggggggcggagcctatggaaaaacgccagcaacgcggcctttttacggttcctggccttttgctggccttttgctcacatgttctttcctgcgttatcccctgattctgtggataaccgtattaccgcctttgagtgagctgataccgctcgccgcagccgaacgaccgagcgcagcgagtcagtgagcgaggaagcggaagagcgc |

| | |
|---|---|
| pCT anti-GFP FspI 30 | cgcctctccccgcgcgttggccgattcattaatgcagctggcacgacaggtttcccgactggaaagcgggcagtgagcgcaacgcaattaatgtgagttacct<br>cactcattaggcaccccaggctttacactttatgcttccggctcctatgttgtgtggaattgtgagcggataacaatttcacacaggaaacagctatgaccatgatt<br>acgccaagctgccagatctgcagccgctatatatccgcggattaacgcgctagccggctgggccgtcgaacggaattaaccctcactaaagggaacaaaa<br>gctgggtacccgacaggttatcagcaacaacacagtcatatccattctcaattagctctaccacagtgtgtgaaccaatgtatccagcaccacctgtaaccaaa<br>acaattttagaagtactttcactttgtaactgagctgtcatttatattgaattttcaaaaattcttacttttttttggatggacgcaaagaagtttaataatcatattacatgg<br>cattaccaccatatacatatccatatacatatccatatctaatcttacttatatgttgtggaaatgtaaagagccccattatcttagcctaaaaaaaccttctctttgga<br>actttcagtaatacgcttaactgctcattgctatattgaagtacggattagaagccgccgagcgggtgacagccctccgaaggaagactctcctccgtgcgtcct<br>cgtcttcaccggtcgcgttcctgaaacgcagatgtgcctcgcgccgcactgctccgaacaataaagattctacaatactagcttttatggttatgaagaggaaaa<br>attggcagtaacctggccccacaaaccttcaaatgaacgaatcaaattaacaaccataggatgataatgcgattagttttttagccttatttctggggtaattaatc<br>agcgaagcgatgatttttgatctattaacagatatataaatgcaaaaactgcataaccactttaactaatactttcaacattttcggtttgtattacttcttattcaaatgt<br>aataaaagtatcaacaaaaaattgttaatatacctctatactttaacgtcaaggagaaaaaaccccggatcgaattccctacttcatacattttcaattaagatgc<br>agttacttcgctgtttttcaatattttctgttattgcttcagttttagcacaggaactgacaactatatgcgagcaaatcccctcaccaactttagaatcgacgccgtact<br>ctttgtcaacgactactattttggccaacgggaaggcaatgcaaggagtttttgaatattacaaatcagtaacgtttgtcagtaattgcggttctcacccctcaacaa<br>ctagcaaaggcagccccataaacacacagtatgtttttaaggacaatagctcgacgattgaaggtagatacccatacgacgttccagactacgctctgcaggc<br>tagtggtggcggaggttctggtggaggcgggtctggtgggggaggatctgccatggtgcagctggtggaaagcggtggtgcactggtgcagccaggtggca<br>gcctgcgtctgagctgcgcggcgagcggctttccggtgaaccgctatagcatgcgctggtatcgtcaggcgccgggcaaagaacgcgaatgggtggcgggc<br>atgagcagcgcgggcgatcgcagcagctatgaagatagcgtgaaaggccgctttaccattagccgcgatgatgcgcgcaacaccgtgtatctgcagatgaa<br>cagcctgaaaccggaagataccgcggtgtattattgcaacgtgaacgtgggctttgaatattggggccagggcacccaggtgaccgtgagcagcgcggccg<br>ctagcgaaaccccaggtacgagcgaaagcgcgaccccggaacatatggtgccgaccatcgtgatggtggacgcctacaagcgttacaagtaatagctcga<br>gatctgataacaacagtgtagatgtaacaaaatcgactttgttcccactgtacttttagctcgtacaaaatacaatatacttttcatttctccgtaaacaacatgttttcc<br>catgtaatatccttttctatttttcgttccgttaccaactttacacatactttatatagctattcacttctatacactaaaaaactaagacaattttaattttgctgcctgccat<br>atttcaatttgttataaattcctataatttatcctattagtagctaaaaaaagatgaatgtgaatcgaatcctaagagaattgag**CCTCAGCAGGTGGCC**<br>**GATTCATTAATGCAGTTTGCGCAGGTGGCCGATTCATTAATGCAGTTTGCTGAGG**ctccaattcgccctatagtgagtc<br>gtattacaattcactggccgtcgttttacaacgtcgtgactgggaaaaccctggcgttacccaacttaatcgccttgcagcacatccccctttcgccagctggcgta<br>atagcgaagaggcccgcaccgatcgccctttcccaacagttgggcagcctgaatggcgaatggacgcgccctgtagcggcgcattaagcgcggcgggtgtg<br>gtggttacgcgcagcgtgaccgctacacttgccagcgccctagcgcccgctcctttcgctttcttcccttcctttctcgccacgttcgccggctttccccgtcaagctc<br>taaatcgggggctccctttagggttccgatttagtgctttacggcacctcgaccccaaaaaacttgattagggtgatggttcacgtagtgggccatcgccctgata<br>gacggtttttcgccctttgacgttggagtccacgttctttaatagtggactcttgttccaaactggaacaacactcaaccctatctcggtctattcttttgatttataaggg<br>attttgccgatttcggcctattggttaaaaaatgagctgatttaacaaaaatttaacgcgaattttaacaaaatattaacgcttacaatttcctgatgcggtattttctcct<br>tacgcatctgtgcggtatttcacaccgcatagatcggcaagtgcacaaacaatacttaaataaatactactcagtaataacctatttcttagcatttttgacgaaattt<br>gctattttgttagagtcttttacaccatttgtctccacacctccgcttacatcaacaccaataacgccatttaatctaagcgcatcaccaacattttctggcgtcagtcc<br>accagctaacataaaatgtaagctttcggggctctcttgccttccaacccagtcagaaatcgagttccaatccaaaagttcacctgtcccacctgcttctgaatca<br>aacaagggaataaacgaatgaggtttctgtgaagctgcactgagtagtatgttgcagtcttttggaaatacgagtcttttaataactggcaaaccgaggaactctt<br>ggtattcttgccacgactcatctccatgcagttggacgatatcaatgccgtaatcattgaccagagccaaaacatcctccttaggttgattacgaaacacgccaa<br>ccaagtatttcggagtgcctgaactatttttatatgcttttacaagacttgaaattttccttgcaataaccgggtcaattgttctctttctattgggcacacatataataccc<br>agcaagtcagcatcggaatctagagcacattctgcggcctctgtgctctgcaagccgcaaactttcaccaatggaccagaactacctgtgaaattaataacag<br>acatactccaagctgcctttgtgtgcttaatcacgtatactcacgtgctcaatagtcaccaatgccctccctcttggccctctccttttcttttttcgaccgaattaattctt<br>aatcggcaaaaaaagaaaagctccggatcaagattgtacgtaaggtgacaagctatttttcaataaagaatatcttccactactgccatctggcgtcataactgc<br>aaagtacacatatattacgatgctgtctattaaatgcttcctatattatatatatagtaatgtcgtttatggtgcactctcagtacaatctgctctgatgccgcatagttaa<br>gccagccccgacacccgccaacacccgctgacgcgccctgacgggcttgtctgctcccggcatccgcttacagacaagctgtgaccgtctccgggagctgc<br>atgtgtcagaggttttcaccgtcatcaccgaaacgcgcgagacgaaagggcctcgtgatacgcctatttttataggttaatgtcatgataataatggtttcttagga<br>cggatcgcttgcctgtaacttacacgcgcctcgtatcttttaatgatggaataatttgggaatttactctgtgtttatttatttttatgttttgtatttggattttagaaagtaaat<br>aaagaaggtagaagagttacggaatgaagaaaaaaaaataaacaaaggtttaaaaaatttcaacaaaaagcgtactttacatatatatttattagacaagaa<br>aagcagattaaatagatatacattcgattaacgataagtaaaatgtaaaatcacaggattttcgtgtgtggtcttctacacagacaagatgaaacaattcggcatt |

| | |
|---|---|
| | aatacctgagagcaggaagagcaagataaaaggtagtatttgttggcgatccccctagagtcttttacatcttcggaaaacaaaaactattttttctttaatttcttttttactttctatttttaatttatatatttatattaaaaaatttaaattataattatttttatagcacgtgatgaaaaggacccaggtggcacttttcggggaaatgtgcgcggaacccctatttgtttatttttctaaatacattcaaatatgtatccgctcatgtcgagacgttgggtgaggttccaactttcaccataatgaaataagatcactaccgggcgtattttttgagttatcgagattttcaggagctaaggaagctaaaatggagaaaaaaatcactggatataccaccgttgatatatcccaatggcatcgtaaagaacattttgaggcatttcagtcagttgctcaatgtacctataaccagaccgttcagctggatattacggcctttttaaagaccgtaaagaaaaataagcacaagttttatccggcctttattcacattcttgcccgcctgatgaatgctcacccggagttccgtatggcaatgaaagacggtgagctggtgatatgggatagtgttcacccttgttacaccgttttccatgagcaaactgaaacgttttcatcgctctggagtgaataccacgacgatttccggcagtttctacacatatattcgcaagatgtggcgtgttacggtgaaaacctggcctatttccctaaagggtttattgagaatatgtttttcgtctcagccaatccctgggtgagtttcaccagttttgatttaaacgtggctaatatggacaacttcttcgcccccgttttcacgatgggcaaatattatacgcaaggcgacaaggtgctgatgccgctggcgattcaggttcatcatgccgtttgtgatggcttccatgtcggcagaatgcttaatgaattacaacagtactgcgatgagtggcagggcggggcgtaatttttttaaggcagttattggtgcccttaaacgcctggtgctacgcctgaataagtgataataagcggatgaatggcagaaattcgaaagcaaattcgacccggtcgtcggttcagggcagggtcgttaaatagccgcttatgtctattgctggtttaccggtttattgactaccggaagcagtgtgaccgtgtgcttctcaaatgcctgaggccagtttgctcaggctctccccgtggaggtaataattgctcgacatgaccaaaatcccttaacgtgagttttcgttccactgagcgtcagaccccgtagaaaagatcaaaggatcttcttgagatcctttttttctgcgcgtaatctgctgcttgcaaacaaaaaaaccaccgctaccagcggtggtttgtttgccggatcaagagctaccaactctttttccgaaggtaactggcttcagcagagcgcagataccaaatactgtccttctagtgtagccgtagttaggccaccacttcaagaactctgtagcaccgcctacatacctcgctctgctaatcctgttaccagtggctgctgccagtggcgataagtcgtgtcttaccgggttggactcaagacgatagttaccggataaggcgcagcggtcgggctgaacggggggttcgtgcacacagcccagcttggagcgaacgacctacaccgaactgagatacctacagcgtgagcattgagaaagcgccacgcttcccgaagggagaaaggcggacaggtatccggtaagcggcagggtcggaacaggagagcgcacgagggagcttccagggggaaacgcctggtatctttatagtcctgtcgggtttcgccacctctgacttgagcgtcgatttttgtgatgctcgtcaggggggccgagcctatggaaaaacgccagcaacgcggcctttttacggttcctggccttttgctggccttttgctcacatgttctttcctgcgttatcccctgattctgtggataaccgtattaccgcctttgagtgagctgataccgctcgccgcagccgaacgaccgagcgcagcgagtcagtgagcgaggaagcggaagagcgcccaatacgcaaac |
| pCT anti-GFP FspI 90 | ccaatacgcaaaccgcctctccccgcgcgttggccgattcattaatgcagctggcacgacaggtttcccgactggaaagcgggcagtgagcgcaacgcaattaatgtgagttacctcactcattaggcaccccaggctttacactttatgcttccggctcctatgttgtgtggaattgtgagcggataacaatttcacacaggaaacagctatgaccatgattacgccaagctgccagatctgcagccgctatatatccgcggattaacgcgctagccggctgggccgtcgaacggaattaaccctcactaaagggaacaaaagctgggtacccgacaggttatcagcaacaacacagtcatatccattctcaattagctctaccacagtgtgtgaaccaatgtatccagcaccacctgtaaccaaaacaattttagaagtactttcactttgtaactgagctgtcatttatattgaattttcaaaaattcttactttttttttggatggacgcaaagaagtttaataatcatattacatggcattaccaccatatacatatccatatacatatccatatctaatcttacttatatgttgtggaaatgtaaagagccccattatcttagcctaaaaaaaccttctctttggaactttcagtaatacgcttaactgctcattgctatattgaagtacggattagaagccgccgagcgggtgacagccctccgaaggaagactctcctccgtgcgtcctcgtcttcaccggtcgcgttcctgaaacgcagatgtgcctcgcgccgcactgctccgaacaataaagattctacaatactagcttttatggttatgaagaggaaaaattggcagtaacctggccccacaaaccttcaaatgaacgaatcaaattaacaaccataggatgataatgcgattagttttttagccttatttctggggtaattaatcagcgaagcgatgatttttgatctattaacagatatataaatgcaaaaactgcataaccactttaactaatactttcaacattttcggtttgtattacttcttattcaaatgtaataaaagtatcaacaaaaaattgttaatatacctctatactttaacgtcaaggagaaaaaaccccggatcgaattccctacttcatacattttcaattaagatgcagttacttcgctgtttttcaatattttctgttattgcttcagttttagcacaggaactgacaactatatgcgagcaaatcccctcaccaactttagaatcgacgccgtactctttgtcaacgactactattttggccaacgggaaggcaatgcaaggagtttttgaatattacaaatcagtaacgtttgtcagtaattgcggttctcacccctcaacaactagcaaaggcagccccataaacacacagtatgtttttaaggacaatagctcgacgattgaaggtagatacccatacgacgttccagactacgctctgcaggctagtggtggcggaggttctggtggaggcgggtctggtgggggaggatctgccatggtgcagctggtggaaagcggtggtgcactggtgcagccaggtggcagcctgcgtctgagctgcgcggcgagcggctttccggtgaaccgctatagcatgcgctggtatcgtcaggcgccgggcaaagaacgcgaatgggtggcgggcatgagcagcgcgggcgatcgcagcagctatgaagatagcgtgaaaggccgctttaccattagccgcgatgatgcgcgcaacaccgtgtatctgcagatgaacagcctgaaaccggaagataccgcggtgtattattgcaacgtgaacgtgggctttgaatattggggccagggcacccaggtgaccgtgagcagcgcggccgctagcgaaaccccaggtacgagcgaaagcgcgaccccggaacatatggtgccgaccatcgtgatggtggacgcctacaagcgttacaagtaatagctcgagatctgataacaacagtgtagatgtaacaaaatcgactttgttcccactgtacttttagctcgtacaaaatacaatatacttttcatttctccgtaaacaacatgttttcccatgtaatatccttttctatttttcgttccgttaccaactttacacatactttatatagctattcacttctatacactaaaaaactaagacaattttaatttgctgcctgccatatttcaatttgttataaattcctataatttatcctattagtagctaaaaaaagatgaatgtgaatcgaatcctaagagaattgag**CCTCAGCAGGGGCATCCCTCCTTTCAAGATAAATAATTTATACACTATTCTATTGGAATCTTAATCATTCTGGCCGATT** |

| | |
|---|---|
| | **CATTAATGCAGTTTGCGCAGGGGCATCCCTCCTTTCAAGATAAATAATTTATACACTATTCTATTGGAATCT<br>TAATCATTCTGGCCGATTCATTAATGCAGTTTGCTGAGG**ctccaattcgccctatagtgagtcgtattacaattcactggccgtcgtttt<br>acaacgtcgtgactgggaaaaccctggcgttacccaacttaatcgccttgcagcacatccccctttcgccagctggcgtaatagcgaagaggcccgcaccga<br>tcgcccttcccaacagttgcggagcctgaatggcgaatggacgcgccctgtagcggcgcattaagcgcggcgggtgtggtggttacgcgcagcgtgaccgct<br>acacttgccagcgccctagcgcccgctcctttcgctttcttcccttcctttctcgccacgttcgccggctttccccgtcaagctctaaatcgggggctccctttagggtt<br>ccgatttagtgctttacggcacctcgaccccaaaaaacttgattagggtgatggttcacgtagtgggccatcgccctgatagacggtttttcgccctttgacgttgga<br>gtccacgttctttaatagtggactcttgttccaaactggaacaacactcaaccctatctcggtctattcttttgatttataagggattttgccgatttcggcctattggttaa<br>aaaatgagctgatttaacaaaaatttaacgcgaattttaacaaaatattaacgcttacaatttcctgatgcggtattttctccttacgcatctgtgcggtatttcacacc<br>gcatagatcggcaagtgcacaaacaatacttaaataaatactactcagtaataacctatttcttagcatttttgacgaaatttgctattttgttagagtcttttacaccat<br>ttgtctccacacctccgcttacatcaacaccaataacgccatttaatctaagcgcatcaccaacattttctggcgtcagtccaccagctaacataaaatgtaagctt<br>tcggggctctcttgccttccaacccagtcagaaatcgagttccaatccaaaagttcacctgtcccacctgcttctgaatcaaacaagggaataaacgaatgagg<br>tttctgtgaagctgcactgagtagtatgttgcagtcttttggaaatacgagtcttttaataactggcaaaccgaggaactcttggtattcttgccacgactcatctccat<br>gcagttggacgatatcaatgccgtaatcattgaccagagccaaaacatcctccttaggttgattacgaaacacgccaaccaagtatttcggagtgcctgaacta<br>tttttatatgcttttacaagacttgaaattttccttgcaataaccgggtcaattgttctctttctattgggcacacatataatacccagcaagtcagcatcggaatctaga<br>gcacattctgcggcctctgtgctctgcaagccgcaaactttcaccaatggaccagaactacctgtgaaattaataacagacatactccaagctgcctttgtgtgct<br>taatcacgtatactcacgtgctcaatagtcaccaatgccctccctcttggccctctccttttcttttttcgaccgaattaattcttaatcggcaaaaaaagaaaagctcc<br>ggatcaagattgtacgtaaggtgacaagctatttttcaataaagaatatcttccactactgccatctggcgtcataactgcaaagtacacatatattacgatgctgtc<br>tattaaatgcttcctatattatatatatagtaatgtcgtttatggtgcactctcagtacaatctgctctgatgccgcatagttaagccagccccgacacccgccaacac<br>ccgctgacgcgccctgacgggcttgtctgctcccggcatccgcttacagacaagctgtgaccgtctccgggagctgcatgtgtcagaggttttcaccgtcatcac<br>cgaaacgcgcgagacgaaagggcctcgtgatacgcctatttttataggttaatgtcatgataataatggtttcttaggacggatcgcttgcctgtaacttacacgcg<br>cctcgtatcttttaatgatggaataatttgggaatttactctgtgtttatttatttttatgttttgtatttggattttagaaagtaaataaagaaggtagaagagttacggaatg<br>aagaaaaaaaaataaacaaaggtttaaaaaatttcaacaaaaagcgtactttacatatatatttattagacaagaaaagcagattaaatagatatacattcgat<br>taacgataagtaaaatgtaaaatcacaggattttcgtgtgtggtcttctacacagacaagatgaaacaattcggcattaatacctgagagcaggaagagcaag<br>ataaaaggtagtatttgttggcgatccccctagagtcttttacatcttcggaaaacaaaaactatttttctttaatttctttttttactttctatttttaatttatatatttatattaa<br>aaaatttaaattataattatttttatagcacgtgatgaaaaggacccaggtggcacttttcggggaaatgtgcgcggaacccctatttgtttatttttctaaatacattca<br>aatatgtatccgctcatgtcgagacgttgggtgaggttccaactttcaccataatgaaataagatcactaccgggcgtattttttgagttatcgagattttcaggagct<br>aaggaagctaaaatggagaaaaaaatcactggatataccaccgttgatatatcccaatggcatcgtaaagaacattttgaggcatttcagtcagttgctcaatgt<br>acctataaccagaccgttcagctggatattacggcctttttaaagaccgtaaagaaaaataagcacaagttttatccggcctttattcacattcttgcccgcctgatg<br>aatgctcacccggagttccgtatggcaatgaaagacggtgagctggtgatatgggatagtgttcacccttgttacaccgttttccatgagcaaactgaaacgttttc<br>atcgctctggagtgaataccacgacgatttccggcagtttctacacatatattcgcaagatgtggcgtgttacggtgaaaacctggcctatttccctaaagggtttat<br>tgagaatatgtttttcgtctcagccaatccctgggtgagtttcaccagttttgatttaaacgtggctaatatggacaacttcttcgcccccgttttcacgatgggcaaat<br>attatacgcaaggcgacaaggtgctgatgccgctggcgattcaggttcatcatgccgtttgtgatggcttccatgtcggcagaatgcttaatgaattacaacagta<br>ctgcgatgagtggcagggcggggcgtaatttttttaaggcagttattggtgcccttaaacgcctggtgctacgcctgaataagtgataataagcggatgaatggc<br>agaaattcgaaagcaaattcgacccggtcgtcggttcagggcagggtcgttaaatagccgcttatgtctattgctggtttaccggtttattgactaccggaagcagt<br>gtgaccgtgtgcttctcaaatgcctgaggccagtttgctcaggctctccccgtggaggtaataattgctcgacatgaccaaaatcccttaacgtgagttttcgttcca<br>ctgagcgtcagaccccgtagaaaagatcaaaggatcttcttgagatcctttttttctgcgcgtaatctgctgcttgcaaacaaaaaaaccaccgctaccagcggt<br>ggtttgtttgccggatcaagagctaccaactctttttccgaaggtaactggcttcagcagagcgcagataccaaatactgtccttctagtgtagccgtagttaggcc<br>accacttcaagaactctgtagcaccgcctacatacctcgctctgctaatcctgttaccagtggctgctgccagtggcgataagtcgtgtcttaccgggttggactca<br>agacgatagttaccggataaggcgcagcggtcgggctgaacggggggttcgtgcacacagcccagcttggagcgaacgacctacaccgaactgagatac<br>ctacagcgtgagcattgagaaagcgccacgcttcccgaagggagaaaggcggacaggtatccggtaagcggcagggtcggaacaggagagcgcacga<br>gggagcttccagggggaaacgcctggtatctttatagtcctgtcgggtttcgccacctctgacttgagcgtcgatttttgtgatgctcgtcaggggggccgagccta<br>tggaaaaacgccagcaacgcggcctttttacggttcctggccttttgctggccttttgctcacatgttctttcctgcgttatcccctgattctgtggataaccgtattaccg<br>cctttgagtgagctgataccgctcgccgcagccgaacgaccgagcgcagcgagtcagtgagcgaggaagcggaagagcgc |

| | |
|---|---|
| PF<br>Nbb102<br>CAM<br>Nick 10 | tggccttttgctcacatgcgcccaatacgcaaaccgcctctccccgcgcgttggccgattcattaatgcaggaatgattaagattccaatagaatagtgtataaatt<br>atttatcttgaaaggagggatgccctggcacgac**CCTCAGCAGGTTTGCTGAGG**cccgactggaaagcgggcagtgagcgcaacgcaatta<br>atgtgagttagctcactcattaggcaccccaggctttacactttatgcttccggctcgtatgttgtgtggaattgtgagcggataacaatttgaattcaaggagacag<br>tcataatgaaatacctattgcctacggcggccgctggattgttattactcgcggcccagccggcaatggcacaggtccagttacaagagtcaggcggggggctt<br>gtccaggccggggggtcactgcgtttatcgtgtgcggcaagtggatacatcagcgacgcttactacatgggatggtatcgccaggcccctgggaaagaacgc<br>gaatttgtggctaccattacccacgggactaacacttactacgcggattccgtaaaagggcgcttcaccatcagccgcgataacgcaaagaacactgtatatct<br>gcaaatgaatagcttaaagcctgaagataccgcggtctattactgtgccgtacttgaaacacgttcttattctttccgctattggggccagggaactcaggtcactg<br>tatcgagccaccatcaccaccatcatggcgcagaacaaaaactcatctcagaagaggatctgtcttaggccgaaactgttgaaagttgtttagcaaaacctcat<br>acagaaaattcatttactaacgtctggaaagacgacaaaactttagatcgttacgctaactatgagggctgtctgtggaatgctacaggcgttgtggtttgtactgg<br>tgacgaaactcagtgttacggtacatgggttcctattgggcttgctatccctgaaaatgagggtggtggctctgagggtggcggttctgagggtggcggttctgag<br>ggtggcggtactaaacctcctgagtacggtgatacacctattccgggctatacttatatcaaccctctcgacggcacttatccgcctggtactgagcaaaacccc<br>gctaatcctaatccttctcttgaggagtctcagcctcttaatactttcatgtttcagaataataggttccgaaataggcagggtgcattaactgtttatacgggcactgtt<br>actcaaggcactgaccccgttaaaacttattaccagtacactcctgtatcatcaaaagccatgtatgacgcttactggaacggtaaattcagagactgcgctttcc<br>attctggctttaatgaggatccattcgtttgtgaatatcaaggccaatcgtctgacctgcctcaacctcctgtcaatgctggcggcggctctggtggtggttctggtgg<br>cggctctgagggtggcggctctgagggtggcggttctgagggtggcggctctgagggtggcggttccggtggcggctccggttccggtgattttgattatgaaaa<br>aatggcaaacgctaataagggggctatgaccgaaaatgccgatgaaaacgcgctacagtctgacgctaaaggcaaacttgattctgtcgctactgattacggt<br>gctgctatcgatggtttcattggtgacgtttccggccttgctaatggtaatggtgctactggtgattttgctggctctaattcccaaatggctcaagtcggtgacggtgat<br>aattcacctttaatgaataatttccgtcaatatttaccttctttgcctcagtcggttgaatgtcgcccttatgtctttggcgctggtaaaccatatgaattttctattgattgtg<br>acaaaataaacttattccgtggtgtctttgcgtttcttttatatgttgccacctttatgtatgtattttcgacgtttgctaacatactgcgtaataaggagtcttaagctagct<br>aacagtcctatgaatcaactacttagatggtattagtgacctgtaacagagcattagcgcaaggtgatttttgacttcttgcgctaatttttgtcatcaaacctgtcgc<br>actccttaatattttgttaaaattcgcgttaaatttttgttaaatcagctcattttttaaccaataggccgaaatcggcaaaatcccttataaatcaaaagaatagaccg<br>agatagggttgagtgttgttccagtttggaacaagagtccactattaaagaacgtggactccaacgtcaaagggcgaaaaaccgtctatcagggcgatggcc<br>cactacgtgaaccatcaccctaatcaagttttttggggtcgaggtgccgtaaagcactaaatcggaaccctaaagggagcccccgatttagagcttgacgggg<br>aaagccggcgaacgtggcgagaaaggaagggaagaaagcgaaaggagcgggcgctagggcgctggcaagtgtagcggtcacgctgcgcgtaacca<br>ccacacccgccgcgcttaatgcgccgctacagggcgcgtcaggtggcacttttcggggaaatgtgcgcggaacccctatttgtttatttttctaaatacattcaaat<br>atgtatccgctcatgtcgagacgttgggtgaggttccaactttcaccataatgaaataagatcactaccgggcgtattttttgagttatcgagattttcaggagctaa<br>ggaagctaaaatggagaaaaaaatcactggatataccaccgttgatatatcccaatggcatcgtaaagaacattttgaggcatttcagtcagttgctcaatgtac<br>ctataaccagaccgttcagctggatattacggcctttttaaagaccgtaaagaaaaataagcacaagttttatccggcctttattcacattcttgcccgcctgatga<br>atgctcacccggagttccgtatggcaatgaaagacggtgagctggtgatatgggatagtgttcacccttgttacaccgttttccatgagcaaactgaaacgttttca<br>tcgctctggagtgaataccacgacgatttccggcagtttctacacatatattcgcaagatgtggcgtgttacggtgaaaacctggcctatttccctaaagggtttatt<br>gagaatatgtttttcgtctcagccaatccctgggtgagtttcaccagttttgatttaaacgtggctaatatggacaacttcttcgcccccgttttcaccatgggcaaata<br>ttatacgcaaggcgacaaggtgctgatgccgctggcgattcaggttcatcatgccgtttgtgatggcttccatgtcggcagaatgcttaatgaattacaacagtact<br>gcgatgagtggcagggcggggcgtaattttttttaaggcagttattggtgcccttaaacgcctggtgctacgcctgaataagtgataataagcggatgaatggcag<br>aaattcgaaagcaaattcgacccggtcgtcggttcagggcagggtcgttaaatagccgcttatgtctattgctggtttaccggtttattgactaccggaagcagtgt<br>gaccgtgtgcttctcaaatgcctgaggccagtttgctcaggctctccccgtggaggtaataattgctcgacatgaccaaaatcccttaacgtgagttttcgttccact<br>gagcgtcagaccccgtagaaaagatcaaaggatcttcttgagatcctttttttctgcgcgtaatctgctgcttgcaaacaaaaaaaccaccgctaccagcggtgg<br>tttgtttgccggatcaagagctaccaactctttttccgaaggtaactggcttcagcagagcgcagataccaaatactgttcttctagtgtagccgtagttaggccacc<br>acttcaagaactctgtagcaccgcctacatacctcgctctgctaatcctgttaccagtggctgctgccagtggcgataagtcgtgtcttaccgggttggactcaag<br>acgatagttaccggataaggcgcagcggtcgggctgaacggggggttcgtgcacacagcccagcttggagcgaacgacctacaccgaactgagatacctac<br>cagcgtgagctatgagaaagcgccacgcttcccgaagggagaaaggcggacaggtatccggtaagcggcagggtcggaacaggagagcgcacgagg<br>gagcttccagggggaaacgcctggtatctttatagtcctgtcgggtttcgccacctctgacttgagcgtcgatttttgtgatgctcgtcaggggggcggagcctatg<br>gaaaaacgccagcaacgcggcctttttacggttcctggccttttgc |

| | |
|---|---|
| PF<br>Nbb102<br>Nick 15 | tggccttttgctcacatgcgcccaatacgcaaaccgcctctccccgcgcgttggccgattcattaatgcaggaatgattaagattccaatagaatagtgtataaatt<br>atttatcttgaaaggagggatgccctgg**CCTCAGCACGACAGGTTTGCTGAGG**cccgactggaaagcgggcagtgagcgcaacgcaatt<br>aatgtgagttagctcactcattaggcaccccaggctttacactttatgcttccggctcgtatgttgtgtggaattgtgagcggataacaatttgaattcaaggagaca<br>gtcataatgaaatacctattgcctacggcggccgctggattgttattactcgcggcccagccggcaatggcacaggtccagttacaagagtcaggcgggggc<br>ttgtccaggccgggggtcactgcgtttatcgtgtgcggcaagtggatacatcagcgacgcttactacatgggatggtatcgccaggcccctgggaaagaacg<br>cgaatttgtggctaccattacccacgggactaacacttactacgcggattccgtaaaagggcgcttcaccatcagccgcgataacgcaaagaacactgtatat<br>ctgcaaatgaatagcttaaagcctgaagataccgcggtctattactgtgccgtacttgaaacacgttcttattctttccgctattggggccagggaactcaggtcac<br>tgtatcgagccaccatcaccaccatcatggcgcagaacaaaaactcatctcagaagaggatctgtcttaggccgaaactgttgaaagttgtttagcaaaacctc<br>atacagaaaattcatttactaacgtctggaaagacgacaaaactttagatcgttacgctaactatgagggctgtctgtggaatgctacaggcgttgtggtttgtact<br>ggtgacgaaactcagtgttacggtacatgggttcctattgggcttgctatccctgaaaatgagggtggtggctctgagggtggcggttctgagggtggcggttctg<br>agggtggcggtactaaacctcctgagtacggtgatacacctattccgggctatacttatatcaaccctctcgacggcacttatccgcctggtactgagcaaaacc<br>ccgctaatcctaatccttctcttgaggagtctcagcctcttaatactttcatgtttcagaataataggttccgaaataggcagggtgcattaactgtttatacgggcact<br>gttactcaaggcactgaccccgttaaaacttattaccagtacactcctgtatcatcaaaagccatgtatgacgcttactggaacggtaaattcagagactgcgcttt<br>ccattctggctttaatgaggatccattcgtttgtgaatatcaaggccaatcgtctgacctgcctcaacctcctgtcaatgctggcggcggctctggtggtggttctggt<br>ggcggctctgagggtggcggctctgagggtggcggttctgagggtggcggctctgagggtggcggttccggtggcggctccggttccggtgattttgattatgaa<br>aaaatggcaaacgctaataagggggctatgaccgaaaatgccgatgaaaacgcgctacagtctgacgctaaaggcaaacttgattctgtcgctactgattac<br>ggtgctgctatcgatggtttcattggtgacgtttccggccttgctaatggtaatggtgctactggtgattttgctggctctaattcccaaatggctcaagtcggtgacggt<br>gataattcacctttaatgaataatttccgtcaatatttaccttctttgcctcagtcggttgaatgtcgcccttatgtctttggcgctggtaaaccatatgaattttctattgatt<br>gtgacaaaataaacttattccgtggtgtctttgcgtttcttttatatgttgccacctttatgtatgtattttcgacgtttgctaacatactgcgtaataaggagtcttaagcta<br>gctaacagtcctatgaatcaactacttagatggtattagtgacctgtaacagagcattagcgcaaggtgatttttgacttcttgcgctaatttttgtcatcaaacctgtc<br>gcactccttaatattttgttaaaattcgcgttaaatttttgttaaatcagctcattttttaaccaataggccgaaatcggcaaaatcccttataaatcaaaagaatagac<br>cgagatagggttgagtgttgttccagtttggaacaagagtccactattaaagaacgtggactccaacgtcaaagggcgaaaaaccgtctatcagggcgatgg<br>cccactacgtgaaccatcaccctaatcaagttttttggggtcgaggtgccgtaaagcactaaatcggaaccctaaagggagcccccgatttagagcttgacgg<br>ggaaagccggcgaacgtggcgagaaaggaagggaagaaagcgaaaggagcgggcgctagggcgctggcaagtgtagcggtcacgctgcgcgtaac<br>caccacacccgccgcgcttaatgcgccgctacagggcgcgtcaggtggcacttttcggggaaatgtgcgcggaacccctatttgtttatttttctaaatacattca<br>aatatgtatccgctcatgtcgagacgttgggtgaggttccaactttcaccataatgaaataagatcactaccgggcgtatttttgagttatcgagattttcaggagct<br>aaggaagctaaaatggagaaaaaaatcactggatataccaccgttgatatatcccaatggcatcgtaaagaacattttgaggcatttcagtcagttgctcaatgt<br>acctataaccagaccgttcagctggatattacggcctttttaaagaccgtaaagaaaaataagcacaagttttatccggcctttattcacattcttgcccgcctgatg<br>aatgctcacccggagttccgtatggcaatgaaagacggtgagctggtgatatgggatagtgttcacccttgttacaccgttttccatgagcaaactgaaacgttttc<br>atcgctctggagtgaataccacgacgatttccggcagtttctacacatatattcgcaagatgtggcgtgttacggtgaaaacctggcctatttccctaaagggtttat<br>tgagaatatgtttttcgtctcagccaatccctgggtgagtttcaccagttttgatttaaacgtggctaatatggacaacttcttcgcccccgttttcaccatgggcaaat<br>attatacgcaaggcgacaaggtgctgatgccgctggcgattcaggttcatcatgccgtttgtgatggcttccatgtcggcagaatgcttaatgaattacaacagta<br>ctgcgatgagtggcagggcggggcgtaattttttaaggcagttattggtgcccttaaacgcctggtgctacgcctgaataagtgataataagcggatgaatggc<br>agaaattcgaaagcaaattcgacccggtcgtcggttcagggcagggtcgttaaatagccgcttatgtctattgctggtttaccggtttattgactaccggaagcagt<br>gtgaccgtgtgcttctcaaatgcctgaggccagtttgctcaggctctccccgtggaggtaataattgctcgacatgaccaaaatcccttaacgtgagttttcgttcca<br>ctgagcgtcagaccccgtagaaaagatcaaaggatcttcttgagatcctttttttctgcgcgtaatctgctgcttgcaaacaaaaaaaccaccgctaccagcggt<br>ggtttgtttgccggatcaagagctaccaactctttttccgaaggtaactggcttcagcagagcgcagataccaaatactgttcttctagtgtagccgtagttaggcc<br>accacttcaagaactctgtagcaccgcctacatacctcgctctgctaatcctgttaccagtggctgctgccagtggcgataagtcgtgtcttaccgggttggactca<br>agacgatagttaccggataaggcgcagcggtcgggctgaacggggggttcgtgcacacagcccagcttggagcgaacgacctacaccgaactgagatac<br>ctacagcgtgagctatgagaaagcgccacgcttcccgaagggagaaaggcggacaggtatccggtaagcggcagggtcggaacaggagagcgcacga<br>gggagcttccagggggaaacgcctggtatctttatagtcctgtcgggtttcgccacctctgacttgagcgtcgatttttgtgatgctcgtcaggggggcggagccta<br>tggaaaaacgccagcaacgcggcctttttacggttcctggccttttgc |
